# Extensive Copy Number Variations in Admixed Indian Population of African Ancestry: Potential Involvement in Adaptation

**DOI:** 10.1093/gbe/evu250

**Published:** 2014-12-10

**Authors:** Ankita Narang, Pankaj Jha, Dhirendra Kumar, Rintu Kutum, Anupam Kumar Mondal, Indian Genome Variation Consortium, Debasis Dash, Mitali Mukerji

**Affiliations:** ^1^G.N. Ramachandran Knowledge Centre for Genome Informatics, Council of Scientific and Industrial Research, Institute of Genomics and Integrative Biology, New Delhi, India; ^2^Genomics and Molecular Medicine, Council of Scientific and Industrial Research, Institute of Genomics and Integrative Biology, New Delhi, India

**Keywords:** CNVs, admixture, African-Indian, population structure, selection, adaptation

## Abstract

Admixture mapping has been enormously resourceful in identifying genetic variations linked to phenotypes, adaptation, and diseases. In this study through analysis of copy number variable regions (CNVRs), we report extensive restructuring in the genomes of the recently admixed African-Indian population (OG-W-IP) that inhabits a highly saline environment in Western India. The study included subjects from OG-W-IP (OG), five different Indian and three HapMap populations that were genotyped using Affymetrix version 6.0 arrays. Copy number variations (CNVs) detected using Birdsuite were used to define CNVRs. Population structure with respect to CNVRs was delineated using random forest approach. OG genomes have a surprising excess of CNVs in comparison to other studied populations. Individual ancestry proportions computed using STRUCTURE also reveals a unique genetic component in OGs. Population structure analysis with CNV genotypes indicates OG to be distant from both the African and Indian ancestral populations. Interestingly, it shows genetic proximity with respect to CNVs to only one Indian population IE-W-LP4, which also happens to reside in the same geographical region. We also observe a significant enrichment of molecular processes related to ion binding and receptor activity in genes encompassing OG-specific CNVRs. Our results suggest that retention of CNVRs from ancestral natives and de novo acquisition of CNVRs could accelerate the process of adaptation especially in an extreme environment. Additionally, this population would be enormously useful for dissecting genes and delineating the involvement of CNVs in salt adaptation.

## Introduction

Copy number variations (CNVs) range in size from 1 kb to several megabases and include deletions, duplications, and large insertions–deletions (indels) (Sebat et al. 2004; Feuk et al. 2006; Redon et al. 2006). CNVs occupy a larger fraction of the human genome in terms of nucleotide sequences when compared with the single-nucleotide polymorphisms (SNPs) (Wain et al. 2009). These have been implicated in many human disorders such as autism, schizophrenia, glioblastoma, and also in phenotypic diversity (Freeman et al. 2006; Beckmann et al. 2007; Cooper et al. 2007; Cook and Scherer 2008; Conrad et al. 2010; Stankiewicz and Lupski 2010). In a few instances, CNVs have also been linked to human adaptations (Iskow et al. 2012). For example, CNV in amylase gene (*AMY1*) has been linked to high starch diet preference (Perry et al. 2007) and indel polymorphism of *APOBEC3b* to differences in malaria susceptibility (Jha et al. 2012). Earlier, in a genome-wide study on large CNVs across 26 Indian populations, we observed population-specific functional enrichments of processes such as serine proteases and their inhibitors, keratinization, and olfactory receptors (Gautam et al. 2012). The structure of CNV in contrast to SNP within a locus is extremely heterogeneous and could generate a diversity of phenotypic outcomes. Because of this, not only its identification and characterization but also its correlation with function is extremely challenging (Alkan et al. 2011; Gautam et al. 2012).

Genomic dissection of admixed populations offers excellent opportunities for mapping disease loci and signature of selection events especially if the admixture is recent, and the parent populations have distinct ancestral history (Darvasi and Shifman 2005; Tang et al. 2007; Basu et al. 2009a, 2009b; Winkler et al. 2010). Many genes implicated in diseases such as focal segmental glomerulosclerosis (Kopp et al. 2008), type 2 diabetes (Duggirala et al. 1999; Goran et al. 2003), and prostate cancer (Freedman et al. 2006) have been identified in African-Americans. However, most of these studies are SNP based, and the involvement of CNVs is relatively under explored. In an earlier study, we have dissected the ancestry of an African-Indian population “OG-W-IP” (OG), also known as Siddi, which resides in a highly saline environment in western India. Our study revealed that the OG derives its ancestry from BantuKenyans and Yorubans from Africa and Indo-European (IE) large populations of north and western part of India. It was estimated that 58.7% of their genomic ancestry was from the African origin (Narang et al. 2011). These findings were also confirmed by similar study conducted by Shah et al. (2011). Functional annotation of ancestry informative markers (AIMs) revealed enrichment of biological processes such as ion-channel activity and cadherins from Indian ancestral populations. Because OGs are migrants and have been exposed to an excessive saline environment, a condition very different from its native environment, we speculated that enrichment of these processes might have been a consequence of selection. As CNVs can have more pronounced effect than SNPs, we felt it would be pertinent to dissect CNVs in this population.

Our study reveals a surprising excess of CNVs in the admixed population when compared with the ancestral Indian and African as well as the HapMap populations. Population structure analysis indicate that the admixed population has evolved its own genomic structure through copy number variable regions (CNVRs) that are unique and common in this population as well as retained from ancestral population that resides in the same habitat. The CNVRs span the entire genome and are significantly enriched in processes related to ion channels and receptor activity. Our results substantiate the hypothesis that CNVRs especially in admixed populations could accelerate the process of adaptation as in this case the admixed population resides in an extremely saline environment, a habitat known to be nonconducive especially for African natives from equatorial regions. Additionally, this population would be enormously useful for dissecting genes and delineating the involvement of CNVs in salt adaptation.

## Materials and Methods

### Population Data Sets and Genotyping

In this study, a total of 152 healthy and unrelated subjects from 6 different Indian populations and 3 populations of HapMap Project (Gibbs et al. 2003) were used. Five Indian populations were represented in the Indian Genome Variation Consortium panel (Indian Genome Variation Consortium 2008; Narang et al. 2010) and an additional population from western part of India (IE-W-LP) was also included in this study. The first data set comprised 62 samples from 5 IE-speaking populations sampled from Northern (N) and Western (W) part of India and are large ethnic groups (LP) and 15 samples of OG. The second data set comprised 75 samples from 3 populations of International HapMap Project: 25 CEU (Utah residents with ancestry from northern and western Europe), 25 LWK (Luhya in Webuye, Kenya), and 25 YRI (Yoruba in Ibadan, Nigeria). Details of sample information and their location are given in the supplementary table S1, Supplementary Material online.

Genotyping of Indian samples were carried out using genome-wide Affymetrix 6.0 SNP array (Affymetrix, Santa Clara, CA). In brief, 250 ng of DNA samples were processed for restriction digestion with Sty1 and Nsp1 separately, following the manufacturer’s recommended protocols. After amplification, both the set of amplified products were pooled and further processed for hybridization and scanning. Genotyping quality was assessed using Affymetrix Genotyping Console Software, and samples having contrast quality control > 0.4 were further used for CNV analysis. HapMap samples genotyped on Affymetrix 6.0 platform (Gibbs et al. 2003; International HapMap 3 Consortium 2010) were considered for this analysis.

### CNV Detection and Construction of CNVR Map

BirdSuite software (v1.5.5) (Korn et al. 2008; McCarroll et al. 2008) was used to call CNVs from combined data sets of Indian and HapMap samples. Canary module from Birdsuite detects copy number states for known copy number polymorphisms (CNPs) using prior information of intensity from 270 HapMap samples. Birdseye, a hidden Markov model-based module of Birdsuite detects additional CNVs other than reported by Canary. Genotype data were also obtained using Birdseed algorithm of Birdsuite package. Further, we used two other CNV calling algorithms—PennCNV (Wang et al. 2007) and Affymetrix Genotyping Console (GTC) version 4.1.4.840 (www.Affymetrix.com) to check the concordance of Birdseye’s CNV calls.

The following filtering criteria were applied for calling CNVs from Birdseye data: 1) only autosomes were considered, 2) number of contiguous probes ≥5, 3) probes less than 10 kb apart, 4) LOD score ≥10, and 5) CNV events of size <1 Mb. Further, in-house Perl script was used for construction of CNVRs by merging overlapping Birdseye’s CNV segments with any base overlap criteria. Additionally, we applied sample frequency threshold of 5% to avoid singletons/rare calls. Frequency matrix of deletion and duplication calls for CNVRs was also generated. Circos (Krzywinski et al. 2009) was used to plot genome-wide distribution of CNV states from CNVR data set across all populations. For data visualization, ggplot2 (Wickham 2009) and circlize packages in R were used. To understand the potential effect of CNVs on SNPs, SNPs within boundaries of both known CNPs and CNVs obtained from Birdseye were extracted using bedtools (Quinlan and Hall 2010).

CNPs and birdseed genotype calls obtained from Birdsuite were also filtered. Genotypes with confidence value <0.1 were retained, and values >0.1 were treated as missing data.

### Population Genetics Analysis

Supervised Random Forest algorithm (Breiman 2001) (implemented in randomForest package of R (Liaw and Wiener 2002) was used to derive proximity among samples based on CNVR and CNP states. We have used 50,000 decision trees to build the model and 23 CNVRs (√total CNVRs) randomly selected at each node in a decision tree. Proximity (dissimilarity) matrix was converted into similarity matrix and visualized using multidimensional scaling (MDS, Qi 2012). All the analysis were performed in R version 3.0.1. For SNP data, we used smartpca program of EIGENSOFT3.0 package (Patterson et al. 2006; Price et al. 2006).

CNV genotype calls for deletions (0 and 1), normal copy (2), and duplications (3 and 4) were converted to allelic states—0/0 (zero copy), 0/1 (one copy), 1/1 (two copies), 1/2 (three copies), and 2/2 (four copies). Biallelic data format was used for population clustering and population differentiation (*F*_ST_) analysis. Population clustering was performed using STRUCTURE 2.3.4 (Pritchard et al. 2000; Falush et al. 2003) with 10,000 burn-in period and 10,000 iterations. Population-specific *F*_ST_ using CNVRs was computed using Arlequin 3.5 (Excoffier et al. 2005). PGDSpider 2.0.5.0 software (Lischer and Excoffier 2012) was used for conversion of genotype data formats required by different population genetics softwares.

### Identification of Ancestry Informative CNVRs in Admixed Population

CNVRs were divided into three subsets: deletions, duplications, and gain–loss on the basis of CNV states in four populations (OG-W-IP, IE-W-LP4, YRI, and LWK). Duplication and deletion subset included 24 and 483 CNVRs, respectively; whereas gain–loss data set had 58 CNVRs. Deletion and duplication CNVR markers were used for further analysis to avoid ambiguity. Ancestry informativeness was computed using frequency of CNVRs in OG and assigned hypothetical Indian (IE-W-LP4) and African putative ancestors (YRI, LWK), as described in our earlier article (Narang et al. 2011). Method of computing ancestry is described in detail in supplementary text S1, Supplementary Material online. Using the criteria, mentioned in supplementary text S1, Supplementary Material online, we binned ancestry into three classes—where CNVRs in OG was 1) close to Indian ancestor(s), 2) close to African ancestor(s), and 3) unique. Further, functional annotation was done for ancestry informative CNVRs (AICs).

### CNVR Annotation and Gene Enrichment Analysis

CNVR data sets were annotated using RefSeq database downloaded from University of California, Santa Cruz table browser. We considered coordinates of longest transcript of a gene if it has multiple isoforms for annotation. Functional enrichment analysis was performed using ToppFun module of ToppGene suite that uses background information from multiple resources (Chen et al. 2009). We used the criteria of *P* value ≤ 0.05 with Bonferroni correction for enrichment analysis.

## Results

### Identification of CNVs

**Fig. 1.—:**
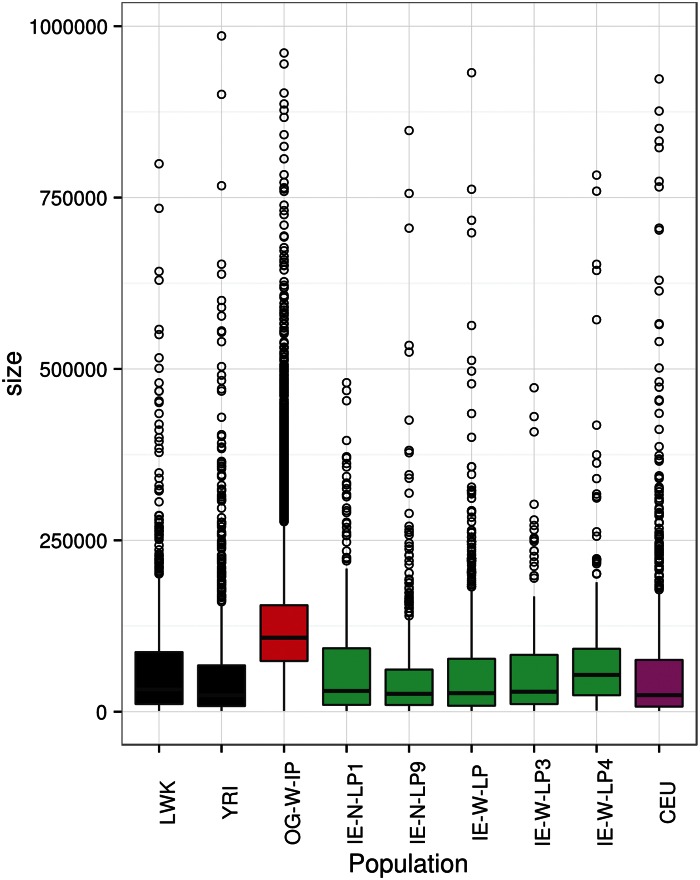
Spectrum of size distribution of CNV segments in the studied populations. Box plots of CNV segment size distribution from African (black), Indian (green), admixed OG-W-IP (red), and CEU (pink) populations are represented. Large CNVs were higher in proportion in OG population in comparison to other populations.

**Fig. 2.—:**
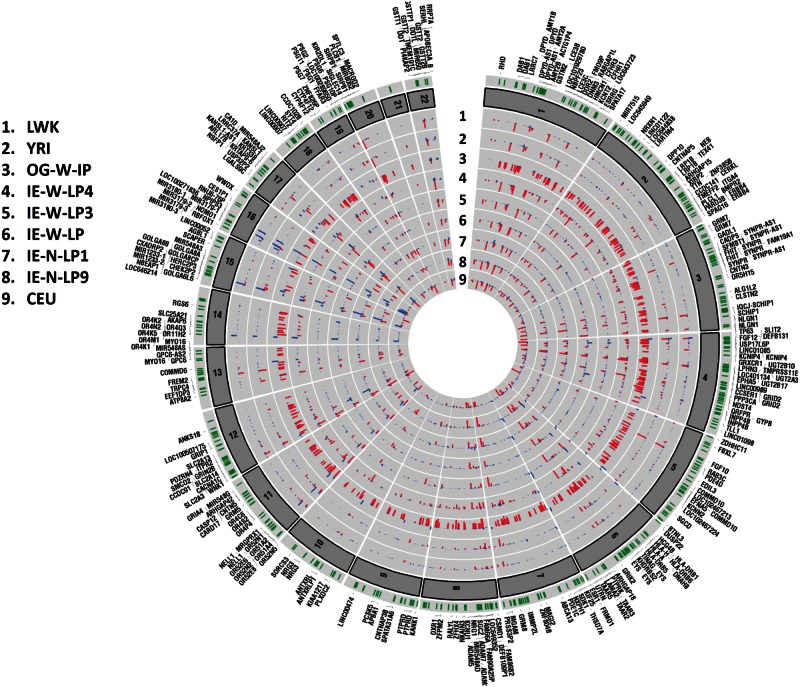
Circular plot showing chromosomal view of CNVRs distribution in studied populations. The outermost circle with vertical green lines represents all the CNVRs from chromosomes 1 to 22. CNVs from different CNVRs in each of the studied populations are represented in the nine concentric circles. Blue- and red-color bars represent duplications and deletion CNV states, respectively. An excess of CNVs across all the autosomal chromosomes in OG population compared with IEs, Africans, and CEU populations is clearly observed.

In this study, 152 individuals of Indian and HapMap populations genotyped on Affymetrix 6.0 were used for CNV analysis. In the previous study using genome-wide SNP data, we had reported that OG population was genetically closer to Bantus of East Africa. Bantus are reported to be closer to Luhya population, another ethnic group in Kenya (Henn et al. 2011). As Luhya is represented in HapMap, we used this population as a surrogate for BantuKenyans for CNV analysis. In the entire data set, a total of 20,023 autosomal CNV calls comprising 17,492 deletions and 2,531 duplications and events were observed. Higher numbers of deletions than duplications have also been reported in several studies (Redon et al. 2006; Conrad et al. 2010; Mills et al. 2011) This has been attributed in-part to bias of genotyping arrays for detecting more number of deletions than duplications (Alkan et al. 2011; Pinto et al. 2011). To confirm our observations, we also checked for the concordance of CNV calls using two other softwares—PennCNV and Affymetrix GTC. Overall, 98.96% of CNV calls from Birdseye software were in agreement with both the softwares (supplementary fig. S1, Supplementary Material online). Average number of CNV calls in OG was significantly higher than observed in Indian and HapMap populations (supplementary table S2, Supplementary Material online). Frequency distribution of CNV events binned into different segment sizes ranging from 1 kb to ≥500 kb revealed CNVs of smaller size to be more frequent (fig. 1 and supplementary fig. S2, Supplementary Material online). Compared with other populations, the segment size of CNVs in OG was much larger (fig. 1). In total, 0.56% of OG genome was under CNVs, whereas fraction of genome covered by CNVs in Indian large populations was only 0.01%. The higher proportion of OG genome under CNVs was consistent with our earlier study based on Affymetrix 50 k array. Though the trends were similar to our earlier study, the estimates were much higher earlier. This could be ascribed to the low-resolution SNP array used for CNV detection (Gautam et al. 2012). Further, a data set of 567 CNVRs was constructed using entire CNVs, and after RefSeq gene annotation, 335 genes were observed to underline structural variation (supplementary table S3, Supplementary Material online). OG population encompasses larger number of CNV events compared with the other populations; especially deletion CNV events are overrepresented (fig. 2). This set of CNVRs spans 0.015% of human genome. This CNVR data set was used for investigating population structure, clustering, and functional annotation of AIMs.

### Stratification and Clustering of Populations Using CNVRs

**Fig. 3.—:**
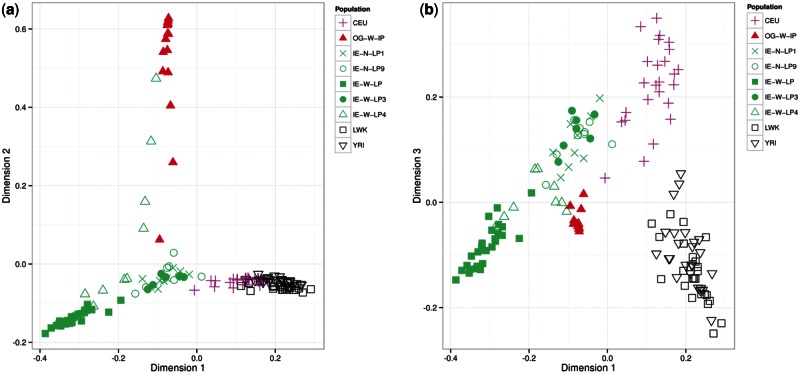
Population structure of studied populations based on MDS. (*a*) MDS analysis of IE large Populations from North and West India, OG, and HapMap populations using 567 CNVRs. OG is closer to IE-W-LP4 compared with other Indian and African populations. (*b*) However, it is important to note that African and Europeans get resolved in the third dimension.

Using a set of 872,188 autosomal SNPs from Affymetrix 6.0 platform, we revalidated our earlier observation of distinctness of OG population as it comes in a perfect cline between Indian (Large populations of Northern and Western India) and African (East and Central West Africa) populations, and there is almost no contribution from European ancestry (supplementary fig. S3, Supplementary Material online). As discussed in the Materials and Methods section, we carried out a random forests approach to compute distance matrix using CNVR data followed by MDS to visualize population stratification. The predicted accuracy of the random forest model is about 81%, and error rate of the model was described by confusion matrix, where comparison between actual and predicted population labels was made (supplementary table S4, Supplementary Material online). MDS analysis reveals that OG individuals cluster separately from African and Indian populations and is close to one of the Indian populations (IE-W-LP4) residing in same geographical region (fig. 3a). This observation was surprising with respect to our previous study based on SNP markers where we reported OG individuals to be closer to African population compared with Indian populations. It seems plausible that this population has acquired CNVs that makes it closer to the population that resides in the same geographical region. Noteworthy, the separation between CEU and African populations was resolved along first and third dimension, whereas no separation was observed in first two dimensions (fig. 3a and 3b). We had same observations from population structure dissected using known CNPs obtained from Canary module of Birdsuite (supplementary fig. S4, Supplementary Material online). To understand the potential effect of CNV markers on SNPs, 22,472 SNP markers within CNV boundaries were also used for population stratification analysis. Population stratification using SNPs within CNV markers also had different spectrum with respect to genome-wide markers but similar to what we observed using CNVRs (supplementary fig. S5, Supplementary Material online). We checked the consistency of the population structure and clustering revealed by SNPs within CNV boundaries by randomly selecting the SNP markers of approximately same data size. Population structure using random data sets was same as reflected by genome-wide marker data sets (supplementary fig. S6, Supplementary Material online).

**Fig. 4.—:**
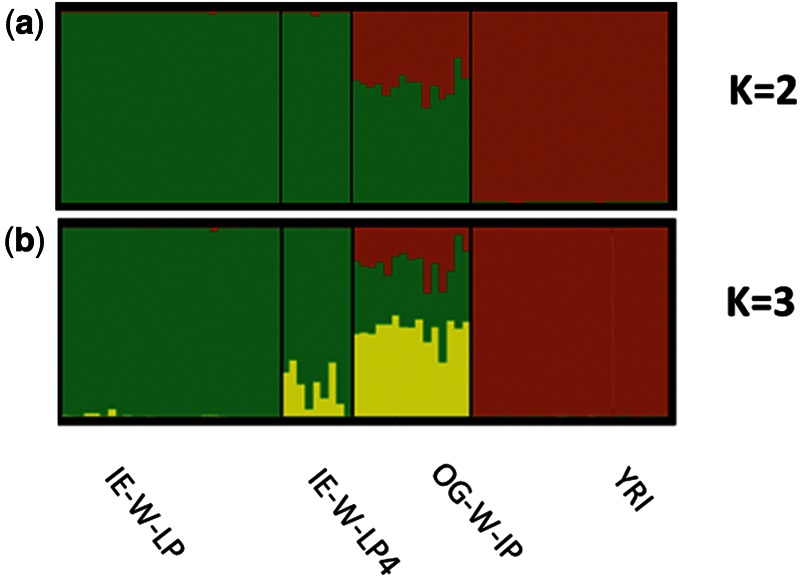
Estimates of individual ancestry proportions of OGs individuals using STRUCTURE. At *k* = 2, analysis based on 22,472 SNP markers within CNV regions revealed that admixed OGs shares major ancestry contribution from Indian ancestors (green) rather than Africans (brown). This observation is in contrast to what is observed with genome-wide SNP markers. At *k* = 3, unique/third component (yellow) accounts for major ancestry proportions (46%) for OG and ancestry contributions of Indian (green) and African ancestors (brown) were 34% and 20%, respectively. Only IE-W-LP4, which is geographical proximal to OG, shares ancestry from third component.

We used STRUCTURE to estimate individual-wise ancestry proportions of OG population using CNVR genotypes. Representative Indian (IE-W-LP4 and IE-W-LP) and African (YRI) populations were chosen for clustering. At *k* = 2, population structure of OGs was not resolved as ancestral populations were not distinctive (supplementary fig. S7*a*, Supplementary Material online). However, at *k* = 3, we observed separation of ancestral populations as expected and a third component contributes maximally (76%) to the ancestry of admixed OG individuals (supplementary fig. S7*b* and table S5, Supplementary Material online). To quantify and analyze the effect of CNVs on admixed populations at fine resolution, we have used SNP markers within CNVs to compute admixture proportions. At *k* = 2, ancestry contribution from Indian populations was higher (0.61%) in comparison to African populations (0.38%) (fig. 4a, supplementary table S6, Supplementary Material online). This finding was contrasting with our previous study using genome-wide SNPs, where proportion of ancestry estimated for African and Indian populations was approximately 60% and approximately 40%, respectively. However, at *k* = 3, contribution of third ancestry component (0.46%) was higher for OG when SNPs within CNVs were used (fig. 4b). This is concordant to ancestry estimates obtained using CNVR genotypes.

As OGs have very recently settled in the Indian subcontinent from Africa, deviation in population structure and excess of unique ancestry proportions in OG individuals through CNVs could be indicative of their role in selection (Cooper et al. 2007).

### Population Differentiation Analysis

**Fig. 5.—:**
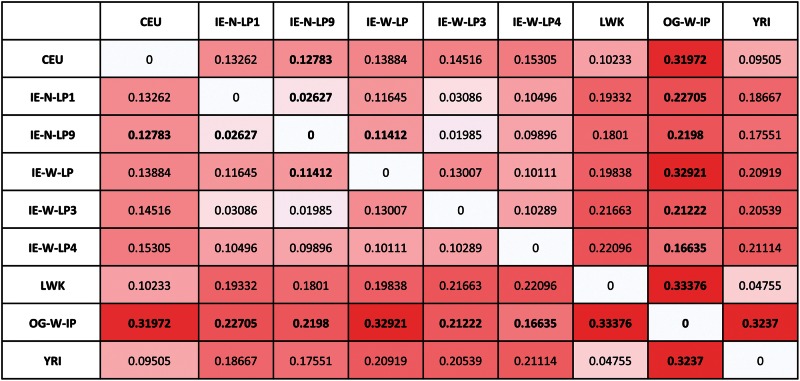
Extent of genetic differentiation between OG and ancestral Indian and African populations. The pairwise FST analysis reveals more closeness of OG with IE west population compared with African and CEU population. This observation is consistent with our clustering analysis.

Pairwise *F*_ST_ distances were computed using CNVRs between OG and ancestral populations. It was observed that *F*_ST_ between OG and IE-W-LP4 was minimum (0.16) compared with other studied Indian and African populations (fig. 5)*.*This genetic closeness can be attributed to geographical proximity and similar environmental/selection pressures operating at the genomic level. Overall, genetic differentiation between OG and Indian populations was less than African populations, whereas IE-W-LP showed high differentiation with OG. The genetic heterogeneity within large Indian populations may be a confounding factor for this high *F*_ST_ observed for IE-W-LP. *F*_ST_ inferences were also in agreement with stratification and clustering analysis. High *F*_ST_ estimates of OG with both Indian and African populations again indicated unique ancestry component in OG as a contribution from CNVs.

### Functional Annotation of AICs

**Fig. 6.—:**
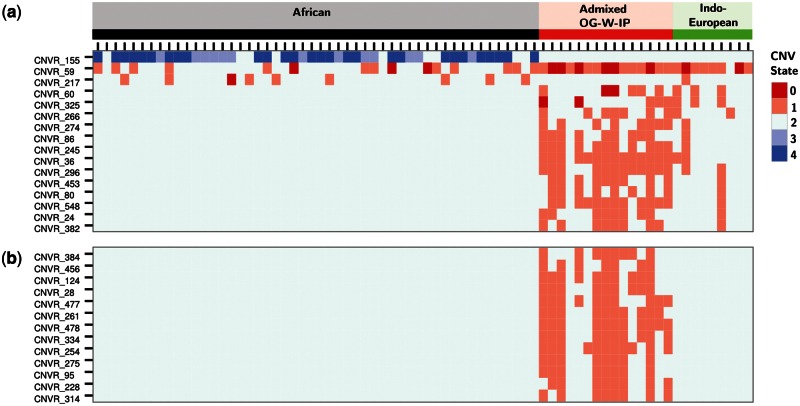
Comparison of CNV spectrum from functionally enriched processes using AICs in OGs. The heatmap represents different states of CNVRs encompassing genes from significantly enriched molecular processes (*a*) in cases where OG is close to Indian ancestor (IE-W-LP4) in comparison to African ancestors (*b*) exclusively in OG. The CNV states are represented by different colors.

We looked for functional enrichment of genes in OG from both Indian and African ancestors. Based on a criteria mentioned in supplementary text S1, Supplementary Material online, there were 194 and 53 unique CNVRs that were close to Indian and African ancestries, respectively. Genic CNVRs in these categories were used for functional enrichment analysis based on different annotations categories in ToppFun. Genes that were close to Indian ancestry were significantly enriched in molecular processes related to signaling receptor activity and glutamate receptor activity (table 1). Pathway enrichment suggests that genes closer to Indian ancestors converge to pathways related to immunology and olfaction. Spectrum of CNV states of enriched genes from different molecular processes also revealed closeness of Indian ancestry to OGs (fig. 6a). There was no enrichment from genes of African ancestry.

**Table 1:** Functional Enrichment Analysis of CNVR Encompassing Genes, Where OG Is Closer to Indian Counterpart (IE-W-LP4)

Category	Name	*P*	Hit Count in Query List	Hit Count in Genome	Hit in Query List
GO: molecular function	Receptor activity	2.06E-07	21	1,617	*ERBB4, OR4N4, ESRRG, DDR2, OR5H15, GRIA4, GRID2, GRM5, GRM7, LRP1B, OR52N1, OR4M2, TINAG, PTPRD, OR52N2, OR52E8, OR52E6, OR52N5, KIR2DL1, KIR2DL4,* and *KIR3DL1*
GO: molecular Function	Glutamate receptor activity	2.85E-06	4	27	*GRIA4, GRID2, GRM5,* and *GRM7*
GO: molecular Function	Transmembrane signaling receptor activity	3.25E-06	17	1,288	*ERBB4, OR4N4, DDR2, OR5H15, GRIA4, GRID2, GRM5, GRM7, OR52N1, OR4M2, PTPRD, OR52N2, OR52E8, OR52E6, OR52N5, KIR2DL4,* and *KIR3DL1*
Pathway	Antigen processing and presentation	1.23E-05	5	82	*LOC100287534, KIR2DL1, KIR2DL4, KIR2DS4,* and *KIR3DL1*
Pathway	Immunoregulatory interactions between a lymphoid and a nonlymphoid cell	2.53E-05	5	95	*ITGA4, KIR2DL1, KIR2DL4, KIR2DS4,* and *KIR3DL1*
Pathway	GPCR downstream signaling	1.29E-04	12	959	*PDE4D, OR4N4, OR5H15, GRM5, GRM7, OR52N1, OR4M2, OR52N2, OR52E8, OR52E6, OR52N5,* and *PLCB1*
Pathway	Natural killer cell-mediated cytotoxicity	1.36E-04	5	135	*LOC100287534, KIR2DL1, KIR2DL4, KIR2DS4,* and *KIR3DL1*
Pathway	Olfactory signaling pathway	1.43E-04	8	425	*OR4N4, OR5H15, OR52N1, OR4M2, OR52N2, OR52E8, OR52E6,* and *OR52N5*

OG population harbors a large number of CNVRs that are unique and not represented in any of the ancestral populations. There are 233 such CNVRs, out of these in 197 regions, there were no CNVs reported in any of the studied population (supplementary fig. S8, Supplementary Material online). These CNVRs were considered as OG specific. These regions had enrichment of molecular processes from genes related to trace-amine receptor activity and calcium ion binding activity for OG-specific CNVRs (table 2). Enriched pathways from OG-specific genes suggest their potential involvement in synaptic and neuronal activities (fig. 6b).

**Table 2:** Functional Enrichment Analysis of Genes in OG-Specific CNVRs

Category	Name	*P*	Hit Count in Query List	Hit Count in Genome	Hit in Query List
GO: molecular function	Trace-amine receptor activity	2.10E-06	3	7	*TAAR2, TAAR3,* and *TAAR5*
GO: molecular Function	Calcium ion binding	1.75E-05	12	694	*FREM2, EYS, PPP3CA, HMCN1, NRXN1, CADPS, CLSTN2, PLCB4, PLCL1, TTN, UTRN,* and *ITPR2*
Pathway	Glutamatergic synapse	1.70E-05	6	116	*PPP3CA, GRIK2, PLCB4, GRIN2B, GRM8,* and *ITPR2*
Pathway	Alzheimer’s disease	3.44E-05	5	79	*CASP12, PPP3CA, PLCB4, GRIN2B,* and *ITPR2*
Pathway	Signaling by NGF	5.19E-05	8	284	*PCSK5, NRG1, PDE1C, SORCS3, FGF10, VAV3, ITPR2,* and *RIT2*
Pathway	Ephrin A reverse signaling	6.63E-05	2	3	*EFNA5* and *EPHA5*
Pathway	GPCRs, Other	9.69E-05	5	98	*TAAR2, TAAR3, TAAR5, GRM8,* and *LPHN3*

## Discussion

In this study, we analyzed the spectrum of CNVs in the admixed Indo-African population from Western India that we identified recently. The admixture involved genomes from two contrasting geographical regions and ethnicity in an extremely saline environment. Interestingly, earlier analysis of genomic regions using SNPs revealed significant enrichment of ion channel and transporter genes from the Indian counterpart in the OG population (Narang et al. 2011). This had hinted at how a selective environment could shape genomes in the admixed population (Tang et al. 2007). Admixture with native populations can facilitate human adaptations in subjects who move to nonnative environment. This has been recently demonstrated in Tibetan populations who have inhabited high-altitude regions in China (Jeong et al. 2014). Alternatively, retention of ancestral genes in nonnative environment could result in maladaptations in admixed populations. Susceptibility for diseases such as focal segmental glomerulosclerosis, hypertension, and diabetes in admixed African-Americans can be an explanation of maladaptation in response to new habitats (Duggirala et al. 1999; Goran et al. 2003; Freedman et al. 2006; Kopp et al. 2008).OG population has come into existence in the last 200 years (Shah et al. 2011), and it is conceivable that the OG genomes might also have undergone changes in the nonnative environment (Narang et al. 2011). In an earlier CNV analysis on Indian populations, which included the OGs, we reported extensive variability across Indian populations, and many regions under CNVs were enriched in biological processes that could confer phenotypic diversity (Gautam et al. 2012). CNVs have been reported to impact genomes to a larger extent compared with SNPs both in terms of structure and magnitude of expression (Stranger et al. 2007; Mileyko et al. 2008; Henrichsen et al. 2009). CNV analysis in an admixed population especially in a highly selective environment could thus provide important insights into the role of these structural variations in shaping genomes. We carried out an extensive CNV analysis of the OG admixed genomes using high-density genotyping arrays. Our study revealed that large fraction of OG genome is under CNVs, as a result of which, the structure of these genomes seem to be entirely different from their ancestral genomes. Further, analysis of AICs in the genomes provided interesting insights, which are discussed below.

Our analysis using high-density SNPs substantiated our earlier observations that the genome of OG seems to be extensively influenced by CNVs, a feature that is reported in other African admixed populations (International HapMap 3 Consortium 2010). To minimize false positives and identify high-quality calls for further analysis, we applied a number of quality filtering criteria (described in Materials and Methods) including a minimum frequency of 5% for defining the 567 CNVRs. The genetic structure of OG population using CNVRs seems to be unique and strikingly different from its ancestral populations. Ancestry estimates from clustering analysis clearly show that OG has a unique genetic component not shared with any other population. Interestingly, some amount of genetic sharing is observed between OG and IE-W-LP4 both of which reside in the same geographical region. When CNVs or SNPs encompassing CNV regions were considered, OG seemed to be closer to an Indian population residing in the same region, whereas in non-CNV regions, OG was more proximal to the African population. It has been earlier reported that population structures could be substantially distorted with respect to CNVs especially in case of selection (Conrad and Hurles 2007). In fact such alterations could help identify populations that are under selection. OG inhabits an extremely saline environment, a habitat that is not known to be conducive to population of African ancestry. Migration and adaptation of a population that is native to equatorial regions to a completely different environment might require substantial changes at genomic level. It is possible that CNVs in response to such selection pressure might contribute to adaptation. Integral role of CNVs during adaptation in saline environment is also highlighted in studies for plants to cope with salt stress (Ma et al. 2013; Oh et al. 2014).

Recent reports have identified selection for genes in response to new environmental conditions and infections in migrant populations such as African-Americans and Roma (Jin et al. 2012; Laayouni et al. 2014). However, such selection signals were absent in their ancestral populations.

Irrespective of previous ancestries, populations residing in the same geographical region are exposed to similar evolutionary pressures and hence might share genomic regions selected for adaptation. One such example is of salt retention in African populations, an adaptive trait against heat stress (Wilson and Grim 1991; Kaufman and Hall 2003). Variations associated with salt retention decrease in frequency outside Africa, and retention of the ancestral allele has been associated with higher prevalence of hypertension, kidney diseases, etc. in the African-American population (Campbell and Tishkoff 2008). Gene-ontology analysis in OG revealed CNVRs to be abundant in processes such as calcium ion binding and trace-amine receptor activity. Perturbations in calcium ion channel genes can lead to multiple disease conditions such as neurologic, cardiologic, and nephrologic abnormalities (supplementary table S7, Supplementary Material online). For example, association of variations in *TTN* with cardiomyopathy (Herman et al. 2012), *ITPR2* with renal carcinoma (Wu et al. 2011), etc. Interestingly, there was a significant enrichment of trace amine receptor genes (*TAAR2*, *TAAR3*, and *TAAR5*), which are a family of G protein-coupled receptors known to bind endogenous biogenic amines and affect behavioral phenotypes (Keller and Vosshall 2008; Nei et al. 2008). Genes with CNVs were dispersed across all chromosomes ruling out the possibility that such an accumulation of CNVs could be a chance event (supplementary fig. S9, Supplementary Material online). It also complements the observations made by SNP in the earlier study. Interestingly, some of these CNV regions were also shared with the parental population residing in same region. Though in terms of SNPs, the parental population is closer to the other IE population, a distinct set of CNVRs, which it shares with OG seem to differentiate it from IE populations of other geographical regions.

To successfully inhabit a contrasting location, OG seems to have shaped its own genome through extensive CNVs. Although a large number of CNVs have been acquired denovo and are present in multiple individuals in the OG population, some CNVs have been retained from the ancestral Indian population, which together might have allowed the population to adapt to its new habitat. This provides an important genetic resource for mapping/identification of genes that could be involved in salt stress adaptation/maladaptation relevant in many phenotypes and diseases.

## Supplementary Material

Supplementary text S1, tables S1–S7, and figures S1–S9 are available at *Genome Biology and Evolution* online (http://www.gbe.oxfordjournals.org/).

Supplementary Data
