# Supplementary material for: Extensive Copy Number Variations in Admixed Indian Population of African Ancestry: Potential Involvement in Adaptation
Source: Genome Biol Evol. 2014 Dec 10;6(12):3171–81. doi: 10.1093/gbe/evu250 (PMC4986450; doi:10.1093/gbe/evu250)
Supplement: Supplementary Data [file supp_6_12_3171__index.html]

Extensive copy number variations in admixed Indian population of African ancestry: Potential involvement in adaptation — Extensive Copy Number Variations in Admixed Indian Population of African Ancestry: Potential Involvement in Adaptation — Supplementary Data 

# Extensive Copy Number Variations in Admixed Indian Population of African Ancestry: Potential Involvement in Adaptation

## Supplementary Data

files

**Files in this Data Supplement:**

- Supplementary Data - pdf file
