# Supplementary material for: Extensive Copy Number Variations in Admixed Indian Population of African Ancestry: Potential Involvement in Adaptation
Source: Genome Biol Evol. 2014 Dec 10;6(12):3171–81. doi: 10.1093/gbe/evu250 (PMC4986450; doi:10.1093/gbe/evu250)
Supplement: Supplementary Data [file supp_evu250_Supplementary_Information_GBE_R1.pdf]

Text S1: Method of computing ancestry information for CNVRs in OG specific to either of ancestral populations.

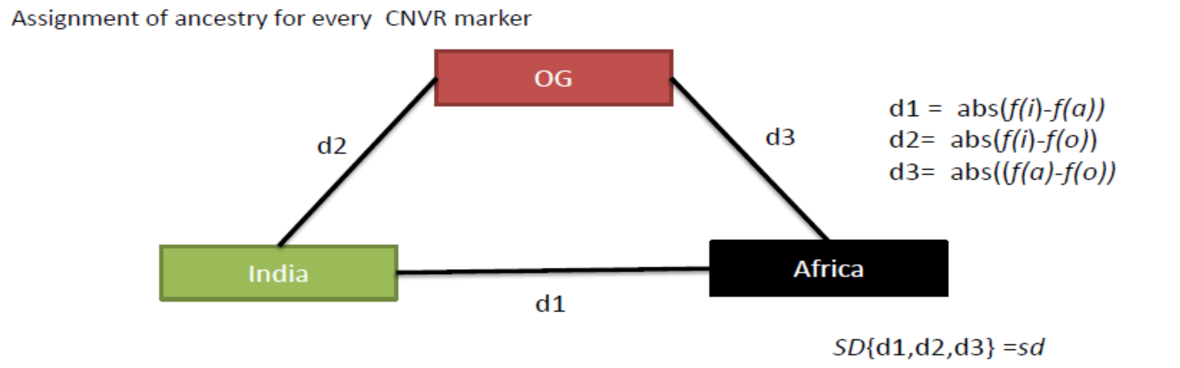

Algorithm for defining ancestry

```
If ((d1 < 0.05) & (sd >=0.05))
{
OG equi-ditsant from both ancestors ;
}
elseif ((d1>0.05)&(d3>d2) & (sd >=0.05))
{
Assign Indian ancestry ;
}
else
{
Assign African Ancestry ;
}}
```

Where
abs= absolute
f(i) = Frequency in putative Indian ancestor(s)
f(a) = Frequency in African ancestor(s)
f(o) = Frequency in OG population
SD= Standard Deviation

Supplementary Figures:

Supplementary Fig.1: Venn diagram showing overlap of Birdseye CNV calls with PennCNV and Affymetrix GTC.

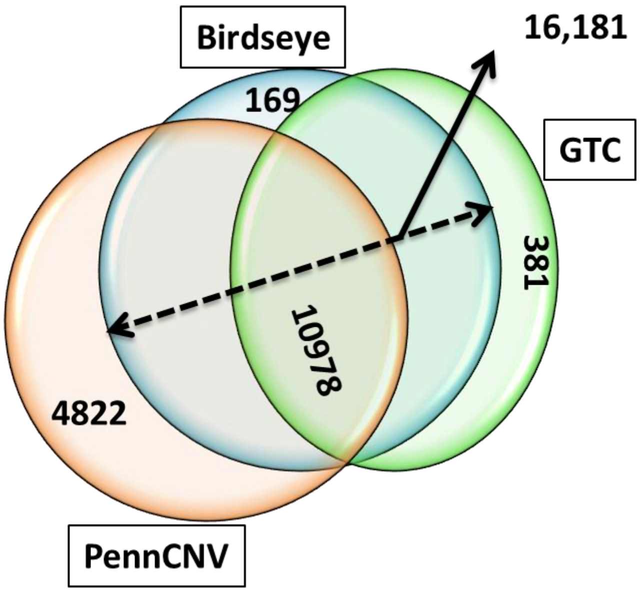

Supplementary Fig.2: Bar plot of CNV counts for deletion and duplications in different size bins in studied populations.

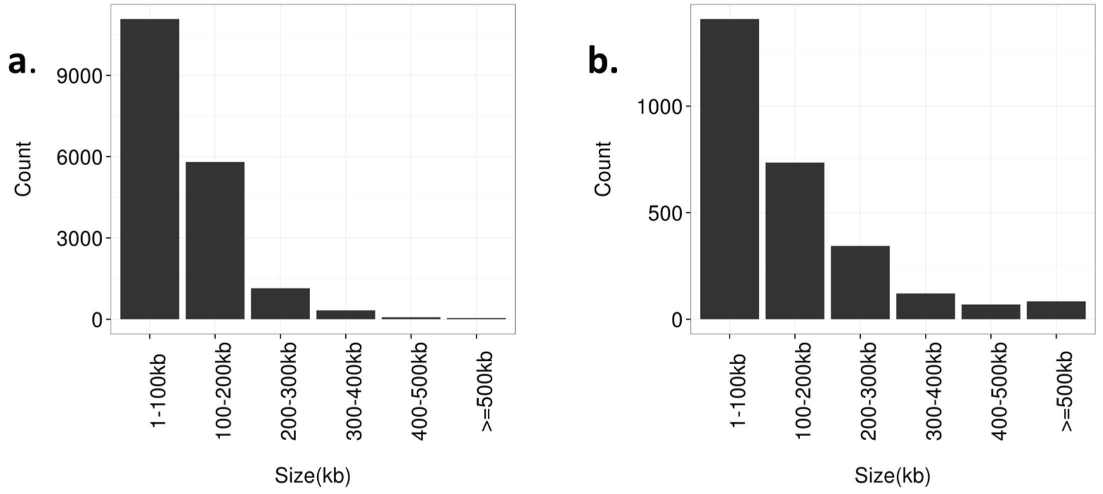

Supplementary Fig.3: Principle component analysis (PCA) of Indian and HapMap Samples based on genome-wide SNP markers.

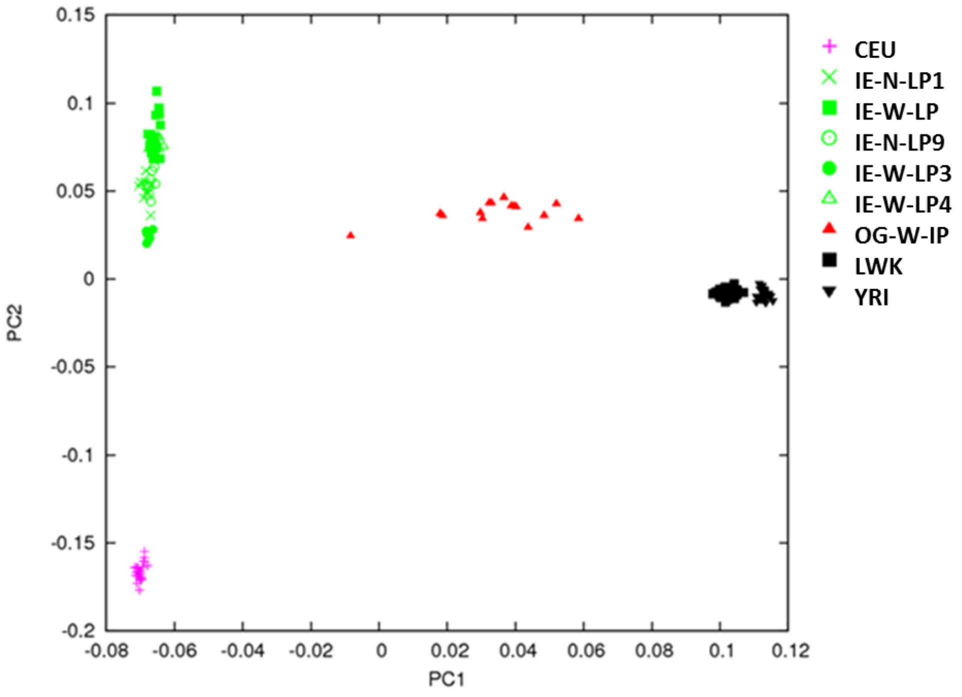

Supplementary Fig.4: Multiple dimensional scaling analysis (MDS) of Indian and HapMap Samples using canary CNV genotype calls.

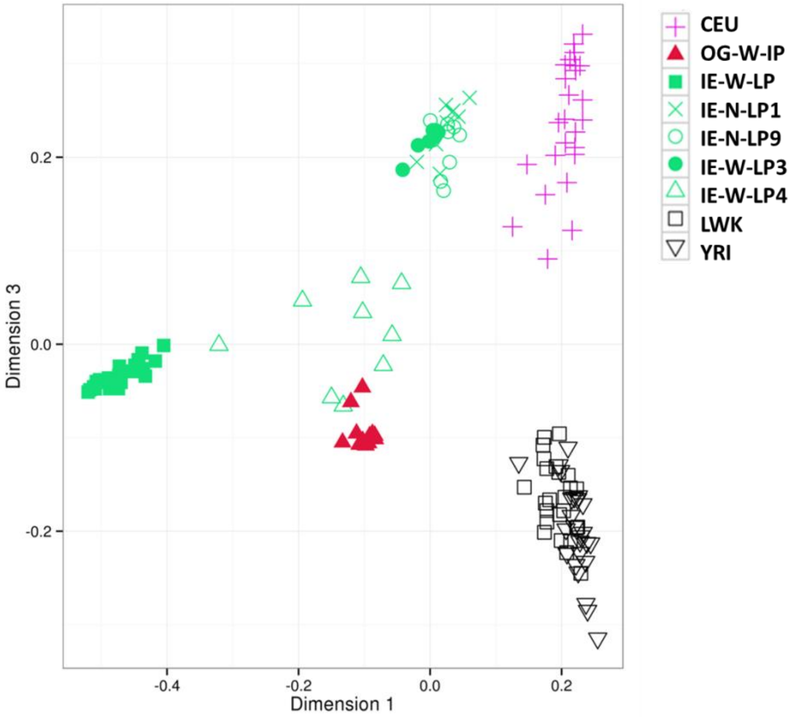

Supplementary Fig.5: Principle component analysis (PCA) of SNPs within CNV boundaries. PCA of SNPs within CNV boundaries further supports the stratification of CNV data.

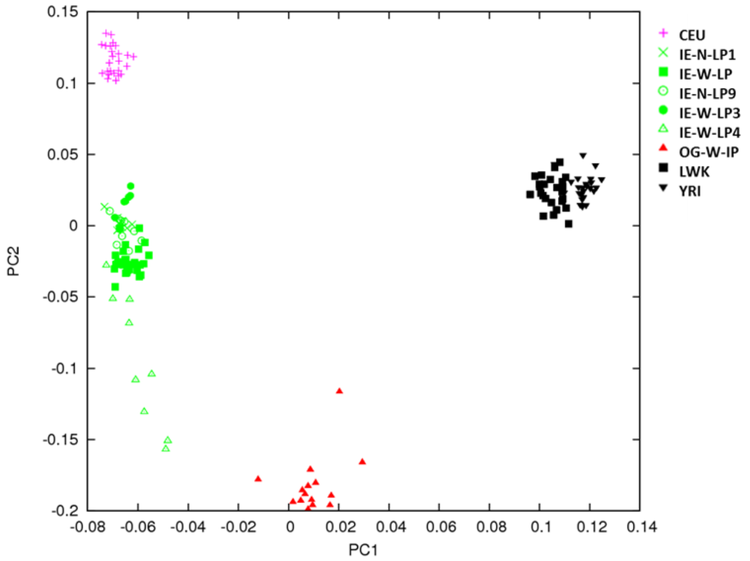

Supplementary Fig.6: Population stratification using two randomly selected datasets of genome-wide SNPs markers.

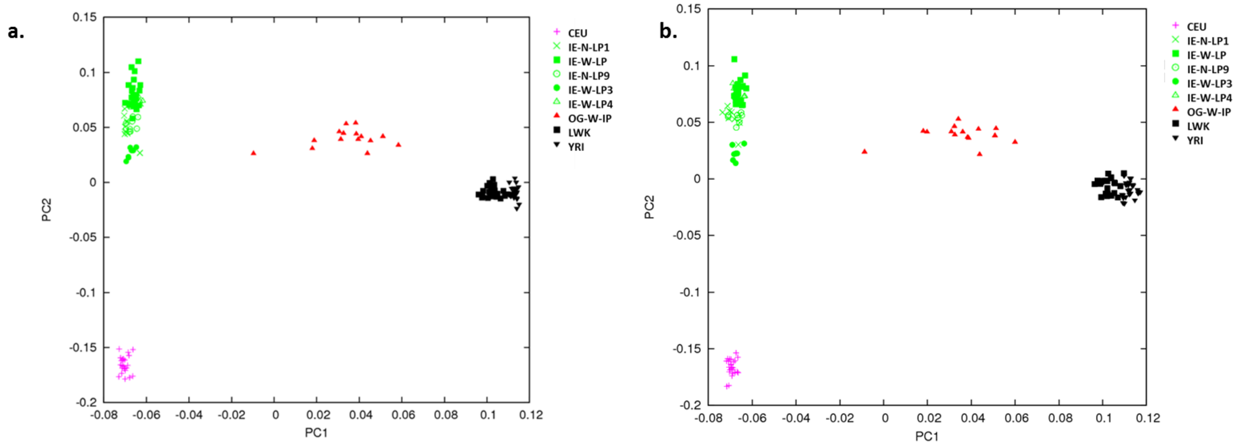

Supplementary Fig.7: Admixture proportions computed using CNVRs.

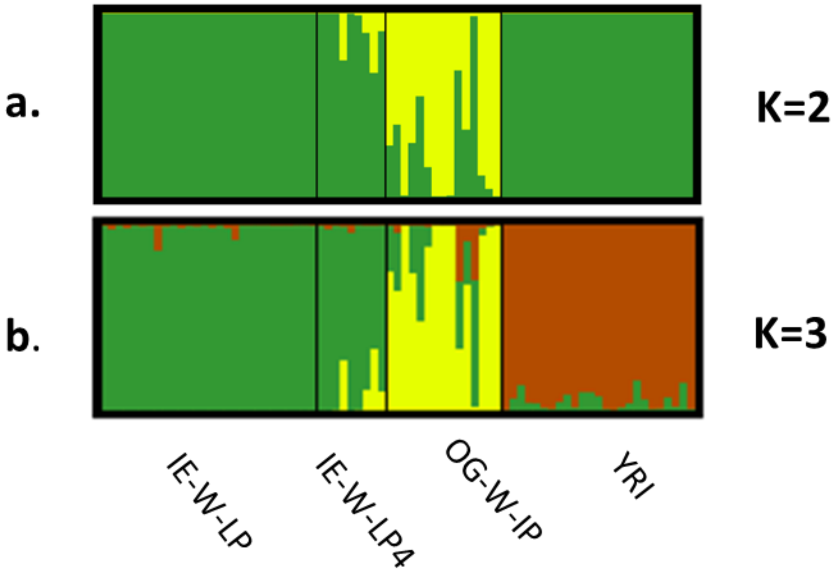

Supplementary Fig.8: Spectrum of CNV states in CNVRs unique to OG population. X-axis represents CNVRs and Y-axis represents individuals from each population.

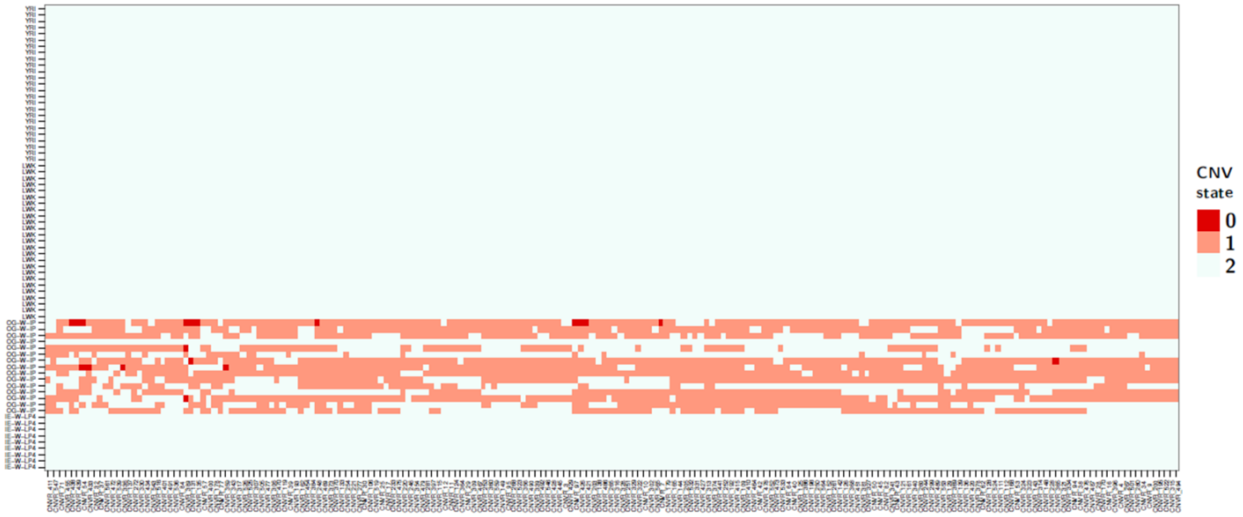

Supplementary Fig.9: Chromosomal distribution of OG specific genes enriched in different molecular processes**.**

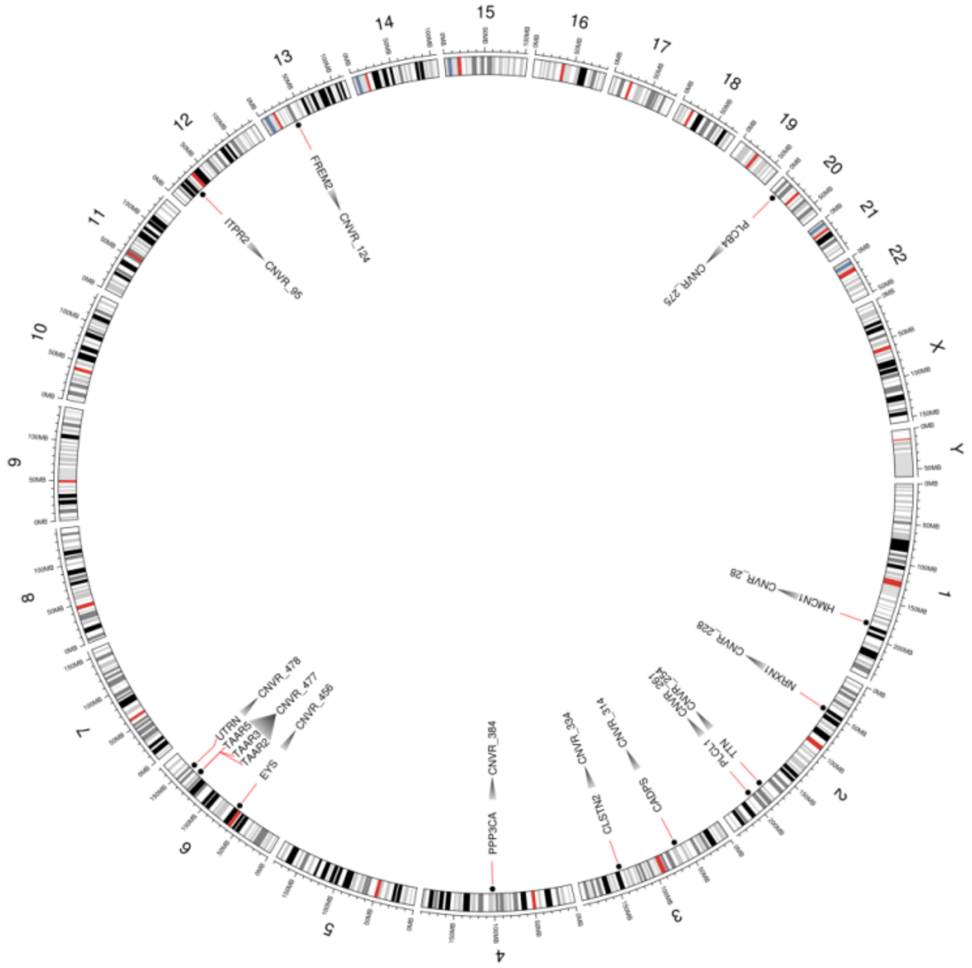

Supplementary Tables:

Supplementary Table1: Details of sample and population information used in the study. Yellow labeled represents samples from Indian populations and green represents from HapMap dataset.

Sample	Population
IGIB_SNP00048_(GenomeWideSNP_6).CEL	**IE-N-LP1**
IGIB_SNP00055_(GenomeWideSNP_6).CEL	**IE-N-LP1**
IGIB_SNP00060_(GenomeWideSNP_6).CEL	**IE-N-LP1**
IGIB_SNP00061_(GenomeWideSNP_6).CEL	**IE-N-LP1**
IGIB_SNP00064_(GenomeWideSNP_6).CEL	**IE-N-LP1**

IGIB_SNP00067_(GenomeWideSNP_6).CEL	**IE-N-LP1**
IGIB_SNP00068_(GenomeWideSNP_6).CEL	**IE-N-LP1**
IGIB_SNP00070_(GenomeWideSNP_6).CEL	**IE-N-LP1**
IGIB_SNP0054_(GenomeWideSNP_6).CEL	**IE-N-LP1**
IGIB_SN00036_(GenomeWideSNP_6).CEL	**IE-N-LP9**
IGIB_SNP00007_(GenomeWideSNP_6).CEL	**IE-N-LP9**
IGIB_SNP00010_(GenomeWideSNP_6).CEL	**IE-N-LP9**
IGIB_SNP00023_(GenomeWideSNP_6).CEL	**IE-N-LP9**
IGIB_SNP00031_(GenomeWideSNP_6).CEL	**IE-N-LP9**
IGIB_SNP00035_(GenomeWideSNP_6).CEL	**IE-N-LP9**
IGIB_SNP00041_(GenomeWideSNP_6).CEL	**IE-N-LP9**
IGIB_SNP00042_(GenomeWideSNP_6).CEL	**IE-N-LP9**
IGIB_SNP00107_(GenomeWideSNP_6).CEL	**IE-N-LP9**
Sample_0010_(GenomeWideSNP_6).CEL	**IE-W-LP**
Sample_0025_(GenomeWideSNP_6).CEL	**IE-W-LP**
Sample_0026_(GenomeWideSNP_6).CEL	**IE-W-LP**
Sample_0030_(GenomeWideSNP_6).CEL	**IE-W-LP**
Sample_0052_(GenomeWideSNP_6).CEL	**IE-W-LP**
Sample_0070_(GenomeWideSNP_6).CEL	**IE-W-LP**
Sample_0088_(GenomeWideSNP_6).CEL	**IE-W-LP**
Sample_0143_(GenomeWideSNP_6).CEL	**IE-W-LP**
Sample_0146_(GenomeWideSNP_6).CEL	**IE-W-LP**
Sample_0149_(GenomeWideSNP_6).CEL	**IE-W-LP**
Sample_0158_(GenomeWideSNP_6).CEL	**IE-W-LP**
Sample_0164_(GenomeWideSNP_6).CEL	**IE-W-LP**
Sample_0014_(GenomeWideSNP_6).CEL	**IE-W-LP**
Sample_0020_(GenomeWideSNP_6).CEL	**IE-W-LP**
Sample_0051_(GenomeWideSNP_6).CEL	**IE-W-LP**
Sample_0054_(GenomeWideSNP_6).CEL	**IE-W-LP**
Sample_0055_(GenomeWideSNP_6).CEL	**IE-W-LP**
Sample_0066_(GenomeWideSNP_6).CEL	**IE-W-LP**
Sample_0103_(GenomeWideSNP_6).CEL	**IE-W-LP**
Sample_0177_(GenomeWideSNP_6).CEL	**IE-W-LP**
Sample_0181_(GenomeWideSNP_6).CEL	**IE-W-LP**
Sample_0186_(GenomeWideSNP_6).CEL	**IE-W-LP**
Sample_0268_(GenomeWideSNP_6).CEL	**IE-W-LP**
Sample_0289_(GenomeWideSNP_6).CEL	**IE-W-LP**
Sample_0291_(GenomeWideSNP_6).CEL	**IE-W-LP**

Sample_0292_(GenomeWideSNP_6).CEL	**IE-W-LP**
Sample_328_(GenomeWideSNP_6).CEL	**IE-W-LP**
Sample_0367_(GenomeWideSNP_6).CEL	**IE-W-LP**
IGIB_SNP02621_(GenomeWideSNP_6).CEL	**IE-W-LP3**
IGIB_SNP02627_(GenomeWideSNP_6).CEL	**IE-W-LP3**
IGIB_SNP02632_(GenomeWideSNP_6).CEL	**IE-W-LP3**
IGIB_SNP02633_(GenomeWideSNP_6).CEL	**IE-W-LP3**
IGIB_SNPO2637_170510_(GenomeWideSNP_6).CEL	**IE-W-LP3**
IGIB_SNPO2638_170510_(GenomeWideSNP_6).CEL	**IE-W-LP3**
IGIB_SNPO2639_170510_(GenomeWideSNP_6).CEL	**IE-W-LP3**
IGIB_SNP_8538_(GenomeWideSNP_6).CEL	**IE-W-LP4**
IGIB_SNP_8540_(GenomeWideSNP_6).CEL	**IE-W-LP4**
IGIB_SNP_8541_(GenomeWideSNP_6).CEL	**IE-W-LP4**
IGIB_SNP-8543_(GenomeWideSNP_6).CEL	**IE-W-LP4**
IGIB_SNP-8544_(GenomeWideSNP_6).CEL	**IE-W-LP4**
IGIB_SNP-8545_(GenomeWideSNP_6).CEL	**IE-W-LP4**
IGIB_SNP-8546_(GenomeWideSNP_6).CEL	**IE-W-LP4**
IGIB_SNP-8547_(GenomeWideSNP_6).CEL	**IE-W-LP4**
IGIB_SNP-8548_(GenomeWideSNP_6).CEL	**IE-W-LP4**
IGIB_SNP_8489_(GenomeWideSNP_6).CEL	**OG-W-IP**
IGIB-SNP-8490_(GenomeWideSNP_6).CEL	**OG-W-IP**
IGIB-SNP-8491_(GenomeWideSNP_6).CEL	**OG-W-IP**
IGIB_SNP_8492_(GenomeWideSNP_6).CEL	**OG-W-IP**
IGIB_SNP_8495_(GenomeWideSNP_6).CEL	**OG-W-IP**
IGIB_SNP_8497_(GenomeWideSNP_6).CEL	**OG-W-IP**
IGIB-SNP-8498_(GenomeWideSNP_6).CEL	**OG-W-IP**
IGIB-SNP-8499_(GenomeWideSNP_6).CEL	**OG-W-IP**
IGIB_SNP_8502_(GenomeWideSNP_6).CEL	**OG-W-IP**
IGIB_SNP_8503_(GenomeWideSNP_6).CEL	**OG-W-IP**
IGIB_SNP_8504_(GenomeWideSNP_6).CEL	**OG-W-IP**
IGIB_SNP_8506_(GenomeWideSNP_6).CEL	**OG-W-IP**
IGIB_SNP_8507_(GenomeWideSNP_6).CEL	**OG-W-IP**
IGIB_SNP_8508_(GenomeWideSNP_6).CEL	**OG-W-IP**
IGIB_SNP_8539_(GenomeWideSNP_6).CEL	**OG-W-IP**
CUPID_p_HapMapPT06_GenomeWideSNP_6_H09_183740.CEL	**CEU**
CUPID_p_HapMapPT06_GenomeWideSNP_6_H11_183772.CEL	**CEU**
CUPID_p_HapMapPT06_GenomeWideSNP_6_H12_183788.CEL	**CEU**
GIGAS_g_GAINmixHapMapAffy2_GenomeWideEx_6_A05_31282.CEL	**CEU**

GIGAS_g_GAINmixHapMapAffy2_GenomeWideEx_6_A06_31298.CEL	**CEU**
GIGAS_g_GAINmixHapMapAffy2_GenomeWideEx_6_A07_31314.CEL	**CEU**
GIGAS_g_GAINmixHapMapAffy2_GenomeWideEx_6_B05_31284.CEL	**CEU**
GIGAS_g_GAINmixHapMapAffy2_GenomeWideEx_6_B08_31332.CEL	**CEU**
GIGAS_g_GAINmixHapMapAffy2_GenomeWideEx_6_C05_31286.CEL	**CEU**
GIGAS_g_GAINmixHapMapAffy2_GenomeWideEx_6_C06_31302.CEL	**CEU**
GIGAS_g_GAINmixHapMapAffy2_GenomeWideEx_6_C07_31318.CEL	**CEU**
GIGAS_g_GAINmixHapMapAffy2_GenomeWideEx_6_C08_31334.CEL	**CEU**
GIGAS_g_GAINmixHapMapAffy2_GenomeWideEx_6_D07_31320.CEL	**CEU**
GIGAS_g_GAINmixHapMapAffy2_GenomeWideEx_6_D08_31336.CEL	**CEU**
GIGAS_g_GAINmixHapMapAffy2_GenomeWideEx_6_E06_31306.CEL	**CEU**
GIGAS_g_GAINmixHapMapAffy2_GenomeWideEx_6_E08_31338.CEL	**CEU**
GIGAS_g_GAINmixHapMapAffy2_GenomeWideEx_6_F05_31292.CEL	**CEU**
GIGAS_g_GAINmixHapMapAffy2_GenomeWideEx_6_G05_31294.CEL	**CEU**
GIGAS_g_GAINmixHapMapAffy2_GenomeWideEx_6_G06_31310.CEL	**CEU**
GIGAS_g_GAINmixHapMapAffy2_GenomeWideEx_6_G07_31326.CEL	**CEU**
GIGAS_g_GAINmixHapMapAffy2_GenomeWideEx_6_G08_31342.CEL	**CEU**
GIGAS_g_GAINmixHapMapAffy2_GenomeWideEx_6_H06_31312.CEL	**CEU**
GIGAS_g_GAINmixHapMapAffy2_GenomeWideEx_6_H08_31344.CEL	**CEU**
NIGHS_p_HapMap_PhIII_Redo_GenomeWideSNP_6_A01_196482.CEL	**CEU**
NIGHS_p_HapMap_PhIII_Redo_GenomeWideSNP_6_A04_196488.CEL	**CEU**
NIGHS_p_HapMap_PhIII_Redo_GenomeWideSNP_6_G06_196636.CEL	**LWK**
NIGHS_p_HapMap_PhIII_Redo_GenomeWideSNP_6_G07_196638.CEL	**LWK**
NIGHS_p_HapMap_PhIII_Redo_GenomeWideSNP_6_G09_196642.CEL	**LWK**
NIGHS_p_HapMap_PhIII_Redo_GenomeWideSNP_6_G10_196644.CEL	**LWK**
NIGHS_p_HapMap_PhIII_Redo_GenomeWideSNP_6_G11_196646.CEL	**LWK**
POSIT_p_HapMapv12_GenomeWideSNP_6_A01_181564.CEL	**LWK**
POSIT_p_HapMapv12_GenomeWideSNP_6_A03_181596.CEL	**LWK**
POSIT_p_HapMapv12_GenomeWideSNP_6_A05_181628.CEL	**LWK**
POSIT_p_HapMapv12_GenomeWideSNP_6_A08_181676.CEL	**LWK**
POSIT_p_HapMapv12_GenomeWideSNP_6_A09_181692.CEL	**LWK**
POSIT_p_HapMapv12_GenomeWideSNP_6_A12_181740.CEL	**LWK**
POSIT_p_HapMapv12_GenomeWideSNP_6_B01_181566.CEL	**LWK**
POSIT_p_HapMapv12_GenomeWideSNP_6_B03_181598.CEL	**LWK**
POSIT_p_HapMapv12_GenomeWideSNP_6_B04_181614.CEL	**LWK**
POSIT_p_HapMapv12_GenomeWideSNP_6_B05_181630.CEL	**LWK**
POSIT_p_HapMapv12_GenomeWideSNP_6_B06_181646.CEL	**LWK**
POSIT_p_HapMapv12_GenomeWideSNP_6_B10_181710.CEL	**LWK**

POSIT_p_HapMapv12_GenomeWideSNP_6_C01_181568.CEL	**LWK**
POSIT_p_HapMapv12_GenomeWideSNP_6_C02_181584.CEL	**LWK**
POSIT_p_HapMapv12_GenomeWideSNP_6_C03_181600.CEL	**LWK**
POSIT_p_HapMapv12_GenomeWideSNP_6_C04_181616.CEL	**LWK**
POSIT_p_HapMapv12_GenomeWideSNP_6_C07_181664.CEL	**LWK**
POSIT_p_HapMapv12_GenomeWideSNP_6_C12_181744.CEL	**LWK**
POSIT_p_HapMapv12_GenomeWideSNP_6_D01_181570.CEL	**LWK**
POSIT_p_HapMapv12_GenomeWideSNP_6_D02_181586.CEL	**LWK**
GIGAS_g_GAINmixHapMapAffy2_GenomeWideEx_6_A01_31218.CEL	**YRI**
GIGAS_g_GAINmixHapMapAffy2_GenomeWideEx_6_A03_31250.CEL	**YRI**
GIGAS_g_GAINmixHapMapAffy2_GenomeWideEx_6_A04_31266.CEL	**YRI**
GIGAS_g_GAINmixHapMapAffy2_GenomeWideEx_6_B02_31236.CEL	**YRI**
GIGAS_g_GAINmixHapMapAffy2_GenomeWideEx_6_B03_31252.CEL	**YRI**
GIGAS_g_GAINmixHapMapAffy2_GenomeWideEx_6_C01_31222.CEL	**YRI**
GIGAS_g_GAINmixHapMapAffy2_GenomeWideEx_6_C04_31270.CEL	**YRI**
GIGAS_g_GAINmixHapMapAffy2_GenomeWideEx_6_D02_31240.CEL	**YRI**
GIGAS_g_GAINmixHapMapAffy2_GenomeWideEx_6_D03_31256.CEL	**YRI**
GIGAS_g_GAINmixHapMapAffy2_GenomeWideEx_6_E01_31226.CEL	**YRI**
GIGAS_g_GAINmixHapMapAffy2_GenomeWideEx_6_E02_31242.CEL	**YRI**
GIGAS_g_GAINmixHapMapAffy2_GenomeWideEx_6_E03_31258.CEL	**YRI**
GIGAS_g_GAINmixHapMapAffy2_GenomeWideEx_6_E04_31274.CEL	**YRI**
GIGAS_g_GAINmixHapMapAffy2_GenomeWideEx_6_F02_31244.CEL	**YRI**
GIGAS_g_GAINmixHapMapAffy2_GenomeWideEx_6_F04_31606.CEL	**YRI**
GIGAS_g_GAINmixHapMapAffy2_GenomeWideEx_6_G01_31230.CEL	**YRI**
GIGAS_g_GAINmixHapMapAffy2_GenomeWideEx_6_G03_31262.CEL	**YRI**
GIGAS_g_GAINmixHapMapAffy2_GenomeWideEx_6_G04_31278.CEL	**YRI**
GIGAS_g_GAINmixHapMapAffy2_GenomeWideEx_6_H02_31248.CEL	**YRI**
GIGAS_g_GAINmixHapMapAffy2_GenomeWideEx_6_H03_31264.CEL	**YRI**
GIGAS_g_GAINmixHapMapAffy2_GenomeWideEx_6_H04_31280.CEL	**YRI**
SCALE_g_GAINmixHapMapAffy1_GenomeWideEx_6_A09_30950.CEL	**YRI**
SCALE_g_GAINmixHapMapAffy1_GenomeWideEx_6_A11_30982.CEL	**YRI**
SCALE_g_GAINmixHapMapAffy1_GenomeWideEx_6_A12_30998.CEL	**YRI**
SCALE_g_GAINmixHapMapAffy1_GenomeWideEx_6_B09_30952.CEL	**YRI**

Supplementary Table 2: Summary statistics of CNV calls identified using Birdseye module in studied populations.

CNV Statistics									
**Population**	**CEU**	**YRI**	**LWK**	**IE-W-LP**	**IE-N-LP1**	**IE-N-LP9**	**IE-W-LP3**	**IE-W-LP4**	**OG-W-IP1**
Sample_Size	25	25	25	28	9	9	7	9	15
Total CNV Calls	1323	1381	969	1099	543	653	310	856	12889
Deletions Count	1058	1061	752	849	381	585	239	714	11853
Duplications Count	265	320	217	241	162	68	71	142	1036
Average CNV Count Per Individual	52.92	55.24	38.76	39.25	60.33	72.56	44.29	95.11	859.27
Average CNV Count Per Individual for Deletions	42.32	42.44	30.08	30.32	37.5	65	34.14	79.33	790.2
Average CNV Count Per Individual for Duplications	10.6	12.8	8.68	8.93	18	7.56	10.14	15.78	69.07
Total Size	83,797,690.00	79,620,667.00	65,977,811.00	64,569,219.00	34,044,675.00	33,880,156.00	18,123,223.00	58,257,235.00	1,628,889,610.00
Average Size	63,339.15	57,654.36	68,088.56	58,752.70	62,697.38	51,883.85	58,462.01	68,057.52	126,378.28
Average Size Del	43,452.77	40,246.41	47,362.16	51,032.06	36,770.70	44,565.28	45,926.28	62,698.61	122,174.00
Average size Dup	142,828.96	115,372.57	139,914.58	84,972.00	123,673.07	114,845.07	100,659.73	95,003.01	174,479.86
Genome fraction*	0.03	0.03	0.02	0.02	0.01	0.01	0.01	0.02	0.57

Supplementary Table 3: Annotated Copy Number Variable Regions (CNVRs) constructed using Indian, OG and HapMap Samples.

CNVR	Chr	Start	Stop	Annotation
CNVR_427	5	1.07E+08	1.07E+08	EFNA5
CNVR_434	5	1.23E+08	1.23E+08	Non-Genic
CNVR_393	4	1.28E+08	1.28E+08	Non-Genic
CNVR_401	4	1.51E+08	1.51E+08	Non-Genic
CNVR_364	4	26878620	26940536	Non-Genic
CNVR_121	13	36887578	36947378	Non-Genic
CNVR_497	7	89459389	89538574	Non-Genic
CNVR_263	2	2.06E+08	2.06E+08	PARD3B
CNVR_346	3	1.75E+08	1.75E+08	NLGN1
CNVR_339	3	1.61E+08	1.61E+08	SCHIP1,IQCJ-SCHIP1
CNVR_228	2	50670383	50735657	NRXN1
CNVR_19	1	1.11E+08	1.11E+08	Non-Genic
CNVR_414	5	46282270	46313969	Non-Genic
CNVR_205	18	61059475	61181260	Non-Genic
CNVR_309	3	36284733	36312767	Non-Genic
CNVR_71	11	37021958	37103169	Non-Genic
CNVR_56	11	4206589	4276290	Non-Genic
CNVR_296	3	7331888	7461254	GRM7
CNVR_108	12	57404846	57474908	Non-Genic
CNVR_92	12	9521823	9622904	Non-Genic
CNVR_174	16	22465433	22612021	LOC653786
CNVR_103	12	38593865	38601020	SLC2A13
CNVR_433	5	1.17E+08	1.17E+08	LOC102467224
CNVR_465	6	82220580	82316176	Non-Genic
CNVR_255	2	1.8E+08	1.8E+08	ZNF385B
CNVR_141	14	25829046	25884384	Non-Genic
CNVR_513	8	5586134	5591903	Non-Genic
CNVR_135	13	90286267	90314977	Non-Genic
CNVR_528	8	40302954	40308668	Non-Genic
CNVR_301	3	22217443	22311827	Non-Genic
CNVR_153	14	85497507	85578803	Non-Genic
CNVR_159	15	28370728	28536721	LOC101059918,CHRFAM7A
CNVR_86	11	1.05E+08	1.05E+08	GRIA4
CNVR_356	4	16705709	16748841	Non-Genic
CNVR_179	16	74664818	74775685	Non-Genic
CNVR_182	17	6047210	6073171	Non-Genic
CNVR_220	2	6594530	6708334	MIR7515

CNVR_529	8	61109299	61170422	Non-Genic
CNVR_5	1	58136669	58167387	DAB1
CNVR_127	13	56656271	56676381	Non-Genic
CNVR_47	10	54584957	54609159	Non-Genic
CNVR_372	4	65502101	65556996	LOC401134
CNVR_20	1	1.12E+08	1.12E+08	Non-Genic
CNVR_304	3	30398317	30406630	Non-Genic
CNVR_535	8	93572450	93636387	Non-Genic
CNVR_247	2	1.45E+08	1.46E+08	TEX41
CNVR_70	11	30671463	30731101	Non-Genic
CNVR_67	11	25565122	25581317	Non-Genic
CNVR_60	11	5817777	5844513	OR52E6,OR52E8
CNVR_555	9	43523459	43740182	SPATA31A6,CNTNAP3B
CNVR_415	5	51948048	52076046	Non-Genic
CNVR_478	6	1.45E+08	1.45E+08	UTRN
CNVR_564	9	1.18E+08	1.18E+08	LINC00474
CNVR_447	5	1.8E+08	1.8E+08	BTNL3
CNVR_455	6	66139833	66162495	EYS
CNVR_397	4	1.44E+08	1.44E+08	INPP4B
CNVR_374	4	66268199	66377522	Non-Genic
CNVR_287	22	21361351	21553233	MIR650
CNVR_54	10	1.07E+08	1.07E+08	SORCS3
CNVR_172	16	19853151	19874863	Non-Genic
CNVR_23	1	1.51E+08	1.51E+08	Non-Genic
CNVR_546	9	7488222	7506684	Non-Genic
CNVR_276	20	10968105	11140548	Non-Genic
CNVR_460	6	77070276	77084501	Non-Genic
CNVR_193	18	23405787	23475185	Non-Genic
CNVR_363	4	21102565	21166574	KCNIP4
CNVR_264	2	2.08E+08	2.08E+08	Non-Genic
CNVR_226	2	40779595	40802273	Non-Genic
CNVR_214	19	48386001	48389173	PSG4
CNVR_522	8	32255842	32359504	NRG1
CNVR_302	3	30157379	30258393	Non-Genic
CNVR_122	13	36970036	36982757	Non-Genic
CNVR_239	2	1.15E+08	1.15E+08	DPP10
CNVR_468	6	87463359	87475703	Non-Genic
CNVR_269	2	2.27E+08	2.27E+08	Non-Genic
CNVR_166	15	85921135	86001598	LINC00052
CNVR_154	15	18652835	19488342	CHEK2P2,LOC646214,CXADRP2,GOLGA8CP,POTEB,POTEB2,CT60,GOLGA6L6,NBEAP1,NF1P2,HERC2P3
CNVR_503	7	1.21E+08	1.21E+08	Non-Genic

CNVR_384	4	1.02E+08	1.02E+08	PPP3CA
CNVR_396	4	1.43E+08	1.43E+08	INPP4B
CNVR_536	8	1.07E+08	1.07E+08	ZFPM2
CNVR_394	4	1.32E+08	1.32E+08	Non-Genic
CNVR_353	4	11951207	12006650	Non-Genic
CNVR_323	3	74602074	74656916	CNTN3
CNVR_446	5	1.79E+08	1.79E+08	Non-Genic
CNVR_402	4	1.53E+08	1.53E+08	Non-Genic
CNVR_343	3	1.65E+08	1.65E+08	Non-Genic
CNVR_110	12	66237533	66282948	LOC100507175
CNVR_482	7	138342	166359	Non-Genic
CNVR_426	5	1.06E+08	1.06E+08	LOC102467213
CNVR_416	5	57361784	57369290	Non-Genic
CNVR_64	11	21213700	21215454	NELL1
CNVR_107	12	57017938	57045691	Non-Genic
CNVR_494	7	77873369	77929599	MAGI2
CNVR_231	2	58911738	59057679	LINC01122
CNVR_104	12	39982173	40103127	PDZRN4
CNVR_119	13	25088798	25160677	ATP8A2
CNVR_57	11	4482950	4515860	Non-Genic
CNVR_545	9	582889	596961	KANK1
CNVR_327	3	1.01E+08	1.01E+08	Non-Genic
CNVR_241	2	1.23E+08	1.23E+08	Non-Genic
CNVR_18	1	1.1E+08	1.1E+08	GSTM1,GSTM2
CNVR_520	8	24412117	24467571	ADAM7
CNVR_78	11	80951189	81093084	Non-Genic
CNVR_181	16	81131034	81140525	Non-Genic
CNVR_567	9	1.21E+08	1.21E+08	Non-Genic
CNVR_464	6	81705925	81748305	Non-Genic
CNVR_487	7	11314044	11390928	THSD7A
CNVR_514	8	12278011	12402552	LOC649352,DEFB109P1,FAM90A25P,FAM66A,FAM86B2,LOC100506990
CNVR_330	3	1.17E+08	1.17E+08	Non-Genic
CNVR_270	20	1505190	1508999	SIRPB1
CNVR_158	15	25593092	25601401	Non-Genic
CNVR_413	5	46197279	46227641	Non-Genic
CNVR_377	4	70155839	70298973	UGT2B28
CNVR_556	9	44667855	44795733	Non-Genic
CNVR_432	5	1.16E+08	1.16E+08	COMMD10
CNVR_461	6	77496587	77509523	Non-Genic
CNVR_310	3	46776821	46824593	Non-Genic
CNVR_124	13	38224255	38326381	FREM2

CNVR_128	13	57303725	57458704	Non-Genic
CNVR_500	7	1.1E+08	1.1E+08	IMMP2L
CNVR_245	2	1.42E+08	1.42E+08	LRP1B
CNVR_171	16	14897340	15013764	MIR3179-1-3,MIR3180-1-3,LOC100288162,MIR6511A-2,MIR6770-2,MIR1972-1,2,NOMO1,NPIPA1,PDXDC1
CNVR_509	7	1.42E+08	1.42E+08	Non-Genic
CNVR_53	10	83876515	83935645	NRG3
CNVR_28	1	1.84E+08	1.84E+08	HMCN1
CNVR_132	13	68150996	68162686	Non-Genic
CNVR_275	20	9162739	9260040	PLCB4
CNVR_48	10	56849322	56850546	Non-Genic
CNVR_8	1	72528701	72583736	Non-Genic
CNVR_39	1	2.37E+08	2.37E+08	Non-Genic
CNVR_452	6	51238911	51323765	Non-Genic
CNVR_11	1	97343743	97493409	DPYD,DPYD-AS1
CNVR_380	4	92680388	92885265	CCSER1
CNVR_331	3	1.2E+08	1.2E+08	Non-Genic
CNVR_338	3	1.54E+08	1.54E+08	Non-Genic
CNVR_527	8	39349340	39506122	ADAM3A,ADAM5
CNVR_382	4	94459846	94551400	GRID2
CNVR_31	1	1.92E+08	1.92E+08	Non-Genic
CNVR_552	9	23553421	23663382	Non-Genic
CNVR_253	2	1.67E+08	1.67E+08	XIRP2
CNVR_354	4	13712387	13714546	Non-Genic
CNVR_125	13	53589768	53626276	Non-Genic
CNVR_389	4	1.16E+08	1.16E+08	NDST4
CNVR_530	8	63579217	63732132	NKAIN3
CNVR_449	6	29948930	30029106	HCG4B,HLA-A,HLA-H
CNVR_170	16	8173425	8272874	Non-Genic
CNVR_456	6	66166301	66186725	EYS
CNVR_373	4	65917164	66020448	EPHA5
CNVR_32	1	1.95E+08	1.95E+08	KCNT2
CNVR_49	10	58179283	58206069	Non-Genic
CNVR_286	22	21048635	21070490	Non-Genic
CNVR_238	2	1.06E+08	1.06E+08	Non-Genic
CNVR_565	9	1.2E+08	1.2E+08	Non-Genic
CNVR_248	2	1.46E+08	1.46E+08	Non-Genic
CNVR_348	3	1.93E+08	1.94E+08	FGF12
CNVR_80	11	88016696	88130554	GRM5
CNVR_69	11	30096711	30141702	Non-Genic
CNVR_203	18	51959733	52007678	Non-Genic
CNVR_368	4	42542587	42670649	GRXCR1

CNVR_280	20	37376195	37537707	Non-Genic
CNVR_324	3	77943364	78111824	Non-Genic
CNVR_258	2	1.85E+08	1.85E+08	Non-Genic
CNVR_562	9	1.04E+08	1.04E+08	Non-Genic
CNVR_366	4	28859054	28920933	Non-Genic
CNVR_347	3	1.91E+08	1.91E+08	TP63
CNVR_131	13	65110021	65118928	Non-Genic
CNVR_553	9	29638921	29745125	Non-Genic
CNVR_436	5	1.44E+08	1.45E+08	Non-Genic
CNVR_111	12	74238449	74284870	Non-Genic
CNVR_114	12	85944878	85990284	Non-Genic
CNVR_106	12	41698809	41833110	Non-Genic
CNVR_559	9	77962279	78005218	PCSK5
CNVR_340	3	1.63E+08	1.63E+08	Non-Genic
CNVR_61	11	18905648	18918939	MRGPRX1
CNVR_512	8	3984471	3991699	CSMD1
CNVR_441	5	1.56E+08	1.56E+08	SGCD
CNVR_303	3	30348828	30393684	Non-Genic
CNVR_265	2	2.13E+08	2.13E+08	ERBB4
CNVR_99	12	29990659	30092681	Non-Genic
CNVR_13	1	1.02E+08	1.02E+08	Non-Genic
CNVR_491	7	46501886	46595634	Non-Genic
CNVR_526	8	36936693	36998013	Non-Genic
CNVR_517	8	14535607	14565222	SGCZ
CNVR_505	7	1.26E+08	1.26E+08	GRM8
CNVR_232	2	59213200	59373373	Non-Genic
CNVR_211	19	20388046	20508229	ZNF826P
CNVR_431	5	1.16E+08	1.16E+08	COMMD10
CNVR_273	20	7186994	7331634	MIR8062
CNVR_164	15	74678296	74682830	SCAPER
CNVR_222	2	16812538	16832397	Non-Genic
CNVR_499	7	1.09E+08	1.09E+08	Non-Genic
CNVR_445	5	1.69E+08	1.69E+08	Non-Genic
CNVR_548	9	9192872	9370781	PTPRD
CNVR_140	14	19227683	19493212	OR4Q3,OR4M1,OR4K5,OR4K1,OR11H2,OR4N2,OR4K2
CNVR_295	3	6626128	6628332	Non-Genic
CNVR_332	3	1.31E+08	1.31E+08	ALG1L2
CNVR_10	1	90602939	90805877	Non-Genic
CNVR_194	18	33746512	33816853	Non-Genic
CNVR_215	19	48394873	48448077	LOC284344,PSG4
CNVR_26	1	1.7E+08	1.7E+08	DNM3

CNVR_467	6	87119599	87218008	Non-Genic
CNVR_306	3	34245407	34388055	Non-Genic
CNVR_180	16	76929941	76942502	WWOX
CNVR_29	1	1.86E+08	1.86E+08	Non-Genic
CNVR_94	12	13682775	13768117	GRIN2B
CNVR_229	2	52607972	52635046	Non-Genic
CNVR_470	6	93205346	93350059	Non-Genic
CNVR_425	5	1.01E+08	1.01E+08	Non-Genic
CNVR_437	5	1.51E+08	1.51E+08	Non-Genic
CNVR_496	7	88423601	88430399	ZNF804B
CNVR_466	6	85204781	85276415	Non-Genic
CNVR_112	12	74321259	74329241	Non-Genic
CNVR_105	12	41543645	41650526	Non-Genic
CNVR_189	17	47327971	47482171	CA10
CNVR_95	12	26419168	26446997	ITPR2
CNVR_259	2	1.89E+08	1.89E+08	Non-Genic
CNVR_457	6	66456203	66460573	EYS
CNVR_365	4	28247468	28318531	Non-Genic
CNVR_42	10	19636901	19756832	Non-Genic
CNVR_66	11	25131608	25240338	Non-Genic
CNVR_351	4	9812560	9949584	Non-Genic
CNVR_274	20	8141308	8217704	PLCB1
CNVR_126	13	55139743	55174769	Non-Genic
CNVR_237	2	89632342	89912071	Non-Genic
CNVR_409	5	15771492	15773597	FBXL7
CNVR_399	4	1.5E+08	1.5E+08	Non-Genic
CNVR_157	15	22225844	22276386	Non-Genic
CNVR_325	3	99367641	99401227	OR5H15
CNVR_246	2	1.44E+08	1.44E+08	ARHGAP15
CNVR_30	1	1.92E+08	1.92E+08	Non-Genic
CNVR_233	2	67225555	67356932	LOC644838
CNVR_202	18	47913179	48083771	Non-Genic
CNVR_554	9	31423095	31576421	Non-Genic
CNVR_290	22	37693565	37705253	APOBEC3A_B
CNVR_390	4	1.19E+08	1.19E+08	Non-Genic
CNVR_55	10	1.1E+08	1.1E+08	Non-Genic
CNVR_359	4	19168772	19213820	Non-Genic
CNVR_163	15	54577995	54579415	Non-Genic
CNVR_113	12	82252960	82457614	Non-Genic
CNVR_221	2	13500735	13574710	Non-Genic
CNVR_74	11	55130608	55210152	OR4P4,OR4S2,OR4C6

CNVR_424	5	1.01E+08	1.01E+08	Non-Genic
CNVR_76	11	80438873	80563413	Non-Genic
CNVR_525	8	36780115	36898000	KCNU1
CNVR_518	8	16177750	16338419	Non-Genic
CNVR_379	4	80715018	80788200	LINC00989
CNVR_21	1	1.47E+08	1.48E+08	NBPF23,LOC101929780
CNVR_400	4	1.5E+08	1.5E+08	Non-Genic
CNVR_266	2	2.13E+08	2.13E+08	ERBB4
CNVR_485	7	9023619	9179513	Non-Genic
CNVR_288	22	22613016	22726814	GSTT2,GSTT2B,DDT,GSTT2,LOC391322,DDTL,GSTT1,GSTTP2,GSTTP1
CNVR_41	10	19103564	19184715	Non-Genic
CNVR_454	6	62553822	62643387	KHDRBS2
CNVR_169	16	7700395	7871589	RBFOX1
CNVR_333	3	1.38E+08	1.39E+08	Non-Genic
CNVR_484	7	8502011	8563426	NXPH1
CNVR_367	4	34462895	34501120	Non-Genic
CNVR_504	7	1.24E+08	1.24E+08	Non-Genic
CNVR_27	1	1.73E+08	1.73E+08	RABGAP1L
CNVR_444	5	1.66E+08	1.66E+08	Non-Genic
CNVR_6	1	58174538	58233385	DAB1
CNVR_345	3	1.75E+08	1.75E+08	NLGN1
CNVR_472	6	94766888	94906565	Non-Genic
CNVR_298	3	18587522	18667097	Non-Genic
CNVR_479	6	1.52E+08	1.52E+08	ESR1
CNVR_547	9	8937937	8984349	PTPRD
CNVR_313	3	59916832	60006571	FHIT
CNVR_149	14	82020078	82074682	Non-Genic
CNVR_12	1	97513711	97660902	DPYD,DPYD-AS1
CNVR_471	6	93630929	93635351	Non-Genic
CNVR_430	5	1.14E+08	1.14E+08	KCNN2
CNVR_285	22	20139185	20175355	TMEM191C,PI4KAP2
CNVR_139	13	1.08E+08	1.08E+08	MYO16
CNVR_212	19	40541333	40553688	FFAR3
CNVR_398	4	1.45E+08	1.45E+08	GYPB
CNVR_524	8	34259121	34356534	Non-Genic
CNVR_209	19	15641343	15660571	CYP4F12
CNVR_168	15	93075077	93084371	Non-Genic
CNVR_419	5	62764844	62766233	Non-Genic
CNVR_488	7	14943958	15049586	Non-Genic
CNVR_387	4	1.13E+08	1.13E+08	Non-Genic
CNVR_284	21	23479938	23547586	Non-Genic

CNVR_463	6	81340908	81346253	Non-Genic
CNVR_541	8	1.35E+08	1.35E+08	Non-Genic
CNVR_256	2	1.82E+08	1.82E+08	CERKL,ITGA4
CNVR_116	12	91395375	91437111	Non-Genic
CNVR_144	14	42291947	42454433	Non-Genic
CNVR_24	1	1.61E+08	1.61E+08	DDR2
CNVR_97	12	28429048	28604607	CCDC91
CNVR_43	10	20505749	20556984	PLXDC2
CNVR_4	1	56470844	56522954	Non-Genic
CNVR_234	2	77049852	77118508	LRRTM4
CNVR_334	3	1.41E+08	1.42E+08	CLSTN2
CNVR_188	17	41521621	42120174	KANSL1-AS1,ARL17B,LRRC37A,ARL17A,LRRC37A2,KANSL1,NSFP1,NSF
CNVR_376	4	69713484	69902857	UGT2A3,UGT2B10
CNVR_408	5	12868780	12873076	Non-Genic
CNVR_411	5	29059061	29090783	Non-Genic
CNVR_282	21	22577635	22586529	Non-Genic
CNVR_448	6	222360	324015	DUSP22
CNVR_156	15	21606530	21610400	Non-Genic
CNVR_217	19	59975655	60040503	KIR2DL1,KIR2DL4,LOC100287534,KIR2DS4,KIR3DL1
CNVR_360	4	19396758	19525219	Non-Genic
CNVR_510	7	1.42E+08	1.42E+08	PRSS3P2
CNVR_34	1	1.98E+08	1.98E+08	Non-Genic
CNVR_316	3	63485004	63496596	SYNPR,SYNPR-AS1
CNVR_89	12	2116123	2128181	CACNA1C
CNVR_540	8	1.35E+08	1.35E+08	Non-Genic
CNVR_196	18	36514418	36519387	Non-Genic
CNVR_200	18	38738369	38849340	RIT2
CNVR_120	13	31430622	31436423	EEF1DP3
CNVR_162	15	44813144	44958601	MIR548A3
CNVR_197	18	36899387	36964001	Non-Genic
CNVR_81	11	94915458	94960751	Non-Genic
CNVR_481	6	1.68E+08	1.68E+08	FRMD1,KIF25
CNVR_300	3	22048394	22139233	Non-Genic
CNVR_508	7	1.41E+08	1.41E+08	MGAM
CNVR_542	8	1.38E+08	1.38E+08	Non-Genic
CNVR_386	4	1.05E+08	1.05E+08	Non-Genic
CNVR_40	10	8737326	8770636	Non-Genic
CNVR_292	3	98317	98526	Non-Genic
CNVR_50	10	59367027	59442553	Non-Genic
CNVR_213	19	47986230	48335218	PSG7,LOC100289650,PSG1,PSG10P,PSG11,PSG2,PSG6

CNVR_279	20	15259370	15265978	MACROD2
CNVR_314	3	62457592	62509509	CADPS
CNVR_560	9	82735266	82784881	Non-Genic
CNVR_147	14	65776943	65841362	Non-Genic
CNVR_278	20	12949715	13091562	SPTLC3
CNVR_267	2	2.15E+08	2.15E+08	SPAG16
CNVR_336	3	1.46E+08	1.46E+08	Non-Genic
CNVR_523	8	32988821	33112493	MIR548AD
CNVR_352	4	10001452	10009766	Non-Genic
CNVR_438	5	1.55E+08	1.55E+08	Non-Genic
CNVR_152	14	84574981	84727510	Non-Genic
CNVR_557	9	71287475	71306626	APBA1
CNVR_297	3	7743672	7942629	GRM7
CNVR_175	16	32274359	32563024	Non-Genic
CNVR_249	2	1.47E+08	1.47E+08	Non-Genic
CNVR_77	11	80946633	80947480	Non-Genic
CNVR_83	11	1E+08	1E+08	MIR548G,ARHGAP42
CNVR_326	3	1E+08	1E+08	Non-Genic
CNVR_242	2	1.24E+08	1.24E+08	Non-Genic
CNVR_261	2	1.99E+08	1.99E+08	PLCL1
CNVR_493	7	76312337	76388137	Non-Genic
CNVR_75	11	80248176	80370189	Non-Genic
CNVR_473	6	1.02E+08	1.03E+08	GRIK2
CNVR_178	16	58193938	58276258	Non-Genic
CNVR_271	20	1509580	1541893	SIRPB1
CNVR_458	6	67575994	67632535	Non-Genic
CNVR_190	17	47873838	48087393	Non-Genic
CNVR_138	13	1.08E+08	1.08E+08	MYO16
CNVR_443	5	1.66E+08	1.66E+08	Non-Genic
CNVR_551	9	13700067	13794580	Non-Genic
CNVR_208	18	67120049	67283060	Non-Genic
CNVR_244	2	1.41E+08	1.42E+08	LRP1B
CNVR_451	6	38944739	38946815	DNAH8
CNVR_558	9	75489452	75614055	Non-Genic
CNVR_489	7	17456985	17534983	Non-Genic
CNVR_519	8	20542037	20570765	Non-Genic
CNVR_25	1	1.65E+08	1.65E+08	FMO9P
CNVR_538	8	1.16E+08	1.16E+08	Non-Genic
CNVR_515	8	13597976	13696093	Non-Genic
CNVR_407	5	751371	878653	ZDHHC11
CNVR_167	15	91608389	91621304	Non-Genic

CNVR_79	11	87211599	87347354	Non-Genic
CNVR_315	3	63387276	63477266	SYNPR,SYNPR-AS1
CNVR_311	3	53003415	53014257	SFMBT1
CNVR_381	4	93628942	93709753	GRID2
CNVR_440	5	1.55E+08	1.55E+08	Non-Genic
CNVR_148	14	71596962	71680672	RGS6
CNVR_223	2	21750697	21928934	LOC645949
CNVR_187	17	36766982	36776021	KRT33B
CNVR_68	11	25663542	25675557	Non-Genic
CNVR_177	16	54353890	54379945	CES1P1
CNVR_511	7	1.55E+08	1.55E+08	Non-Genic
CNVR_224	2	24460486	24464632	Non-Genic
CNVR_563	9	1.05E+08	1.05E+08	Non-Genic
CNVR_33	1	1.95E+08	1.95E+08	CFHR3,CFHR1,CFHR4
CNVR_161	15	35245649	35342552	Non-Genic
CNVR_36	1	2.15E+08	2.15E+08	ESRRG
CNVR_439	5	1.55E+08	1.55E+08	Non-Genic
CNVR_335	3	1.45E+08	1.46E+08	Non-Genic
CNVR_151	14	83213340	83364474	Non-Genic
CNVR_15	1	1.06E+08	1.06E+08	Non-Genic
CNVR_391	4	1.23E+08	1.23E+08	QRFPR
CNVR_14	1	1.04E+08	1.04E+08	ACTG1P4,AMY2A,AMY2B,AMY1A,AMY1B,AMY1C
CNVR_130	13	65094530	65106602	Non-Genic
CNVR_268	2	2.22E+08	2.22E+08	Non-Genic
CNVR_143	14	36402878	36439609	SLC25A21
CNVR_9	1	74144096	74218172	Non-Genic
CNVR_155	15	19764921	20089383	OR4M2,OR4N4,OR4N3P,LOC727924,REREP3
CNVR_283	21	23351419	23355927	Non-Genic
CNVR_369	4	59662156	59696608	Non-Genic
CNVR_236	2	88914239	89189769	Non-Genic
CNVR_532	8	79088587	79255302	Non-Genic
CNVR_206	18	63110187	63118245	Non-Genic
CNVR_490	7	31809999	31983667	PDE1C
CNVR_98	12	28885160	28915801	Non-Genic
CNVR_498	7	1.08E+08	1.09E+08	Non-Genic
CNVR_422	5	83222082	83335815	EDIL3
CNVR_429	5	1.1E+08	1.1E+08	Non-Genic
CNVR_549	9	11931913	11948106	Non-Genic
CNVR_1	1	25465715	25519573	RHD
CNVR_474	6	1.04E+08	1.04E+08	Non-Genic
CNVR_272	20	6455559	6562991	Non-Genic

CNVR_291	22	41227226	41280435	SERHL,RRP7A,SERHL2
CNVR_410	5	20535921	20606744	Non-Genic
CNVR_3	1	55731828	55763020	Non-Genic
CNVR_210	19	15661729	15685943	CYP4F12
CNVR_251	2	1.51E+08	1.52E+08	Non-Genic
CNVR_305	3	30711780	30833631	GADL1
CNVR_459	6	74648953	74658138	Non-Genic
CNVR_357	4	18476305	18552811	Non-Genic
CNVR_385	4	1.04E+08	1.04E+08	Non-Genic
CNVR_235	2	78543001	78575410	Non-Genic
CNVR_137	13	1.03E+08	1.03E+08	MIR548AS
CNVR_195	18	34194091	34356944	Non-Genic
CNVR_82	11	99438533	99586799	CNTN5
CNVR_423	5	90853657	91039700	Non-Genic
CNVR_136	13	93202489	93326700	GPC6-AS2,GPC6
CNVR_184	17	19440328	19478062	Non-Genic
CNVR_230	2	53141525	53289795	Non-Genic
CNVR_186	17	36675211	36684057	KRTAP9-6
CNVR_294	3	5633413	5748145	Non-Genic
CNVR_442	5	1.63E+08	1.63E+08	Non-Genic
CNVR_475	6	1.29E+08	1.29E+08	PTPRK
CNVR_250	2	1.51E+08	1.51E+08	Non-Genic
CNVR_561	9	1.04E+08	1.04E+08	Non-Genic
CNVR_341	3	1.63E+08	1.63E+08	Non-Genic
CNVR_52	10	83758325	83799382	NRG3
CNVR_318	3	64279206	64362799	Non-Genic
CNVR_277	20	12077939	12204779	Non-Genic
CNVR_502	7	1.18E+08	1.18E+08	Non-Genic
CNVR_299	3	21843427	21904234	Non-Genic
CNVR_192	17	66303350	66481942	Non-Genic
CNVR_227	2	49322295	49406398	Non-Genic
CNVR_133	13	71466141	71577801	Non-Genic
CNVR_395	4	1.38E+08	1.38E+08	Non-Genic
CNVR_533	8	79952880	80018924	Non-Genic
CNVR_199	18	38169747	38388326	LINC00907
CNVR_506	7	1.33E+08	1.33E+08	Non-Genic
CNVR_91	12	8449755	8475939	Non-Genic
CNVR_129	13	64202016	64293607	Non-Genic
CNVR_65	11	21486828	21605232	NELL1
CNVR_370	4	62542106	62807317	LPHN3
CNVR_344	3	1.67E+08	1.67E+08	Non-Genic

CNVR_417	5	57992583	57994889	RAB3C
CNVR_495	7	83992228	84130820	Non-Genic
CNVR_476	6	1.3E+08	1.3E+08	LAMA2,ARHGAP18
CNVR_58	11	4924226	4933658	OR51A4,OR51A2
CNVR_312	3	59692188	59727070	FHIT
CNVR_469	6	91532929	91634535	Non-Genic
CNVR_521	8	25030360	25040250	Non-Genic
CNVR_7	1	70197030	70297470	LRRC7
CNVR_405	4	1.73E+08	1.73E+08	Non-Genic
CNVR_362	4	20981101	20985949	KCNIP4
CNVR_207	18	64847351	64904306	CCDC102B
CNVR_123	13	37185007	37262933	TRPC4
CNVR_38	1	2.17E+08	2.17E+08	LOC643723
CNVR_173	16	21319904	21501135	SLC7A5P2,NPIPB3,LOC100271836
CNVR_329	3	1.04E+08	1.04E+08	Non-Genic
CNVR_101	12	33180225	33203464	Non-Genic
CNVR_72	11	38414524	38419763	Non-Genic
CNVR_240	2	1.17E+08	1.17E+08	Non-Genic
CNVR_412	5	44131960	44390890	FGF10
CNVR_85	11	1.04E+08	1.04E+08	CARD17
CNVR_350	4	8979788	9066075	USP17L6P,DEFB131
CNVR_544	8	1.4E+08	1.4E+08	Non-Genic
CNVR_63	11	21143571	21205419	NELL1
CNVR_165	15	85101125	85222377	AGBL1
CNVR_406	4	1.79E+08	1.79E+08	LINC01098
CNVR_87	11	1.24E+08	1.24E+08	Non-Genic
CNVR_566	9	1.2E+08	1.2E+08	Non-Genic
CNVR_115	12	89004082	89117120	Non-Genic
CNVR_219	2	6215763	6225326	Non-Genic
CNVR_307	3	34578323	34628739	Non-Genic
CNVR_118	12	1.13E+08	1.13E+08	Non-Genic
CNVR_453	6	54308667	54482248	TINAG
CNVR_59	11	5739501	5810219	OR52N1,OR52N5,OR52N2
CNVR_378	4	72615741	72702475	SLC4A4
CNVR_145	14	43570867	43662409	Non-Genic
CNVR_337	3	1.49E+08	1.49E+08	Non-Genic
CNVR_483	7	3381423	3418715	SDK1
CNVR_17	1	1.08E+08	1.08E+08	VAV3
CNVR_358	4	18677873	18775940	Non-Genic
CNVR_44	10	20922405	21035317	Non-Genic
CNVR_486	7	11256406	11308516	Non-Genic

CNVR_501	7	1.14E+08	1.14E+08	Non-Genic
CNVR_289	22	23993985	24248709	IGLL3P,MIR6817,LRP5L,CRYBB2P1
CNVR_322	3	70867684	71014006	Non-Genic
CNVR_176	16	33208395	33555980	RNU6-76P
CNVR_100	12	30130534	30134989	Non-Genic
CNVR_403	4	1.62E+08	1.62E+08	Non-Genic
CNVR_216	19	56834164	56840009	SIGLEC14
CNVR_185	17	31464091	31509204	Non-Genic
CNVR_435	5	1.44E+08	1.44E+08	Non-Genic
CNVR_492	7	48570725	48585686	ABCA13
CNVR_293	3	930813	1007142	Non-Genic
CNVR_73	11	49279587	49317264	Non-Genic
CNVR_257	2	1.85E+08	1.85E+08	Non-Genic
CNVR_16	1	1.07E+08	1.07E+08	Non-Genic
CNVR_281	20	37761688	37869101	Non-Genic
CNVR_321	3	68504082	68630961	FAM19A1
CNVR_428	5	1.1E+08	1.1E+08	Non-Genic
CNVR_531	8	77816500	78003228	ZFHX4
CNVR_90	12	7905081	8010135	SLC2A3,SLC2A14
CNVR_462	6	79025784	79116741	Non-Genic
CNVR_371	4	64375574	64390818	Non-Genic
CNVR_218	2	4191739	4201042	Non-Genic
CNVR_51	10	66977929	66984452	Non-Genic
CNVR_355	4	13722031	13895351	LINC01085
CNVR_421	5	73338237	73445948	Non-Genic
CNVR_361	4	20164818	20196278	SLIT2
CNVR_477	6	1.33E+08	1.33E+08	TAAR5,TAAR3,TAAR2
CNVR_418	5	58571441	58625543	PDE4D
CNVR_539	8	1.3E+08	1.3E+08	Non-Genic
CNVR_134	13	75007537	75015769	COMMD6
CNVR_342	3	1.64E+08	1.64E+08	Non-Genic
CNVR_534	8	85406374	85456374	RALYL
CNVR_96	12	27539008	27542629	SMCO2
CNVR_349	3	1.94E+08	1.94E+08	Non-Genic
CNVR_183	17	18296117	18367186	USP32P2,LGALS9C
CNVR_142	14	32165589	32278520	AKAP6
CNVR_150	14	82847656	83013970	Non-Genic
CNVR_392	4	1.25E+08	1.25E+08	Non-Genic
CNVR_2	1	34875241	34877078	Non-Genic
CNVR_375	4	69043083	69188929	TMPRSS11E,UGT2B17
CNVR_252	2	1.52E+08	1.52E+08	NEB

CNVR_420	5	66746319	66848896	Non-Genic
CNVR_102	12	36339406	36473016	Non-Genic
CNVR_45	10	24149834	24252491	KIAA1217
CNVR_117	12	98319424	98322865	ANKS1B
CNVR_191	17	49534732	49884231	MIR548AJ2
CNVR_37	1	2.16E+08	2.16E+08	SPATA17
CNVR_550	9	11957011	11965492	Non-Genic
CNVR_388	4	1.15E+08	1.15E+08	Non-Genic
CNVR_35	1	2.14E+08	2.14E+08	Non-Genic
CNVR_225	2	34552819	34590561	Non-Genic
CNVR_46	10	47011195	47174643	ANTXRL,ANTXRLP1
CNVR_320	3	65151156	65213409	Non-Genic
CNVR_543	8	1.38E+08	1.39E+08	Non-Genic
CNVR_254	2	1.79E+08	1.79E+08	CCDC141,TTN
CNVR_88	12	739370	744290	WNK1
CNVR_93	12	11402844	11434653	Non-Genic
CNVR_198	18	38120204	38132158	LINC00907
CNVR_260	2	1.93E+08	1.93E+08	TMEFF2
CNVR_383	4	97924174	98039314	Non-Genic
CNVR_201	18	39108271	39229064	SYT4
CNVR_62	11	20977136	21062948	NELL1
CNVR_308	3	36115876	36262332	Non-Genic
CNVR_450	6	32562253	32678343	HLA-DRB6,HLA-DRB1,HLA-DRB5
CNVR_480	6	1.68E+08	1.68E+08	Non-Genic
CNVR_204	18	56954529	57081213	Non-Genic
CNVR_262	2	2.03E+08	2.03E+08	BMPR2
CNVR_317	3	63501100	63502905	SYNPR,SYNPR-AS1
CNVR_160	15	32507087	32618236	MIR1233-1,MIR1233-2,GOLGA8B,GOLGA8A
CNVR_319	3	64902065	64959530	ADAMTS9-AS2
CNVR_507	7	1.36E+08	1.36E+08	Non-Genic
CNVR_146	14	53201287	53352059	Non-Genic
CNVR_84	11	1.04E+08	1.04E+08	CASP12
CNVR_328	3	1.04E+08	1.04E+08	Non-Genic
CNVR_243	2	1.25E+08	1.25E+08	CNTNAP5
CNVR_404	4	1.67E+08	1.67E+08	TLL1
CNVR_109	12	65304434	65478528	GRIP1
CNVR_22	1	1.51E+08	1.51E+08	LCE3C,LCE3B
CNVR_537	8	1.07E+08	1.08E+08	OXR1
CNVR_516	8	13843303	13906795	Non-Genic

Supplementary Table 4: Confusion matrix computed using random forest shows error rate in sample classification.

Call: randomForest (formula = Population ~ . data = data ntree = 50000, proximity = TRUE importance = T);<br>Type of random forest: classification;<br>Number of trees: 50000;<br>No. of variables tried at each split: 23;<br>OOB estimate of error rate: 19.08%										
**Confusion matrix:**										
**Original\Predicted**	**CEU**	**IE-W-LP**	**IE-N-LP1**	**IE-N-LP9**	**IE-W-LP3**	**IE-W-LP4**	**LWK**	**OG-W-IP**	**YRI**	**class.error**
**CEU**	24	0	0	0	0	0	1	0	0	0.04
**IE-W-LP**	0	28	0	0	0	0	0	0	0	0
**IE-N-LP1**	0	3	4	1	1	0	0	0	0	0.5555556
**IE-N-LP9**	0	2	1	3	3	0	0	0	0	0.6666667
**IE-W-LP3**	0	2	1	3	1	0	0	0	0	0.8571429
**IE-W-LP4**	0	5	0	0	0	3	0	1	0	0.6666667
**LWK**	0	0	0	0	0	0	24	0	1	0.04
**OG-W-IP**	0	0	0	0	0	2	0	13	0	0.1333333
**YRI**	0	0	0	0	0	0	2	0	23	0.08

Supplementary Table 5: Individual-wise membership proportions estimated by STRUCTURE for OG population using CNVR dataset.

Estimated ancestry proportions of OG individuals using CNVRs

(a.) K=2

**Samples**	**Cluster 1**	**Cluster 2**
OG-W-IP	0.278	0.722
OG-W-IP	0.395	0.605
OG-W-IP	0.005	0.995
OG-W-IP	0.292	0.708
OG-W-IP	0.546	0.454
OG-W-IP	0.16	0.84

(b.) K=3

**Samples**	**Cluster 1 (OG)**	**Cluster2 (IE)**	**Cluster3 (AFR)**
OG-W-IP	0.75	0.25	0.00
OG-W-IP	0.65	0.31	0.04
OG-W-IP	1.00	0.00	0.00
OG-W-IP	0.74	0.26	0.00
OG-W-IP	0.48	0.51	0.00
OG-W-IP	0.88	0.11	0.01

OG-W-IP	0.004	0.996	OG-W-IP	1.00	0.00	0.00
OG-W-IP	0.004	0.996	OG-W-IP	1.00	0.00	0.00
OG-W-IP	0.009	0.991	OG-W-IP	1.00	0.00	0.00
OG-W-IP	0.689	0.311	OG-W-IP	0.33	0.37	0.30
OG-W-IP	0.364	0.636	OG-W-IP	0.68	0.23	0.09
OG-W-IP	0.982	0.018	OG-W-IP	0.02	0.68	0.30
OG-W-IP	0.115	0.885	OG-W-IP	0.95	0.04	0.02
OG-W-IP	0.044	0.956	OG-W-IP	0.99	0.01	0.00
OG-W-IP	0.001	0.999	OG-W-IP	1.00	0.00	0.00
Membership Proportion	**0.25**	**0.74**	Membership Proportion	**0.76**	**0.18**	**0.05**

- **IE denotes Indo-European**
- **AFR denotes Africa**

Supplementary Table 6: Individual-wise membership proportions estimated by STRUCTURE for OG population using SNPs within CNV boundaries.

Estimated ancestry proportions of OG individuals using SNPs within CNVs

(a.) K=2 | **(b.) K=3**

**Sample**	**Cluster 1 (IE)**	**Cluster 2 (AFR)**	**Sample**	**Cluster 1 (IE)**	**Cluster 2 (AFR)**	**Cluster 3 (OG)**
OG-W-IP	0.638	0.362	OG-W-IP	0.38	0.18	0.44
OG-W-IP	0.623	0.377	OG-W-IP	0.35	0.21	0.44
OG-W-IP	0.608	0.392	OG-W-IP	0.35	0.21	0.44
OG-W-IP	0.621	0.379	OG-W-IP	0.33	0.19	0.49
OG-W-IP	0.561	0.439	OG-W-IP	0.26	0.25	0.49
OG-W-IP	0.608	0.392	OG-W-IP	0.31	0.16	0.53
OG-W-IP	0.672	0.328	OG-W-IP	0.39	0.14	0.47
OG-W-IP	0.632	0.368	OG-W-IP	0.36	0.17	0.47
OG-W-IP	0.629	0.371	OG-W-IP	0.32	0.16	0.51
OG-W-IP	0.492	0.508	OG-W-IP	0.25	0.35	0.40
OG-W-IP	0.617	0.383	OG-W-IP	0.34	0.19	0.47
OG-W-IP	0.545	0.455	OG-W-IP	0.37	0.34	0.29
OG-W-IP	0.569	0.431	OG-W-IP	0.26	0.23	0.51
OG-W-IP	0.757	0.243	OG-W-IP	0.50	0.04	0.47
OG-W-IP	0.653	0.347	OG-W-IP	0.37	0.13	0.50
**Membership proportions**	**0.61**	**0.38**	**Membership Proportion**	**0.34**	**0.20**	**0.46**

Supplementary Table 7: Association of OG specific genes in enriched molecular processes with various diseases/traits.

GWAS	
**Genes**	**Disease/Trait**
CADPS	Breast size
CLSTN2	Multiple sclerosis (OCB status)
FREM2	Cytomegalovirus antibody response
HMCN1	QRS duration
ITPR2	Renal cell carcinoma
NRXN1	Metabolite levels (HVA/MHPG ratio)
PLCB4	Neutrophil count
PLCL1	Prostate cancer (gene x gene interaction)
PPP3CA	Corneal structure
TTN	Breast size
**OMIM**	
**Genes**	**Disease**
EYS	Retinitis pigmentosa 25
FREM2	Fraser syndrome
HMCN1	Macular degeneration
NRXN1	Pitt-Hopkins-like syndrome 2
PLCB4	Auriculocondylar syndrome 2
TTN	Cardiomyopathy
